# Circulating cell-specific extracellular vesicles as biomarkers for the diagnosis and monitoring of chronic liver diseases

**DOI:** 10.1007/s00018-022-04256-8

**Published:** 2022-04-10

**Authors:** Lauren A. Newman, Kate Muller, Andrew Rowland

**Affiliations:** 1 Department of Clinical Pharmacology, College of Medicine and Public Health, Flinders University, Adelaide, SA Australia; 2 Department of Gastroenterology and Hepatology, College of Medicine and Public Health, Flinders Medical Centre, Adelaide, SA Australia

**Keywords:** Chronic liver disease, Non-alcoholic fatty liver disease, Metabolic-associated fatty liver disease, Extracellular vesicles, Minimally invasive biomarkers, Tissue-specific biomarkers

## Abstract

Chronic liver diseases represent a burgeoning health problem affecting billions of people worldwide. The insufficient performance of current minimally invasive tools is recognised as a significant barrier to the clinical management of these conditions. Extracellular vesicles (EVs) have emerged as a rich source of circulating biomarkers closely linked to pathological processes in originating tissues. Here, we summarise the contribution of EVs to normal liver function and to chronic liver pathologies; and explore the use of circulating EV biomarkers, with a particular focus on techniques to isolate and analyse cell- or tissue-specific EVs. Such approaches present a novel strategy to inform disease status and monitor changes in response to treatment in a minimally invasive manner. Emerging technologies that support the selective isolation and analysis of circulating EVs derived only from hepatic cells, have driven recent advancements in EV-based biomarker platforms for chronic liver diseases and show promise to bring these techniques to clinical settings.

## Introduction

Chronic liver diseases represent a significant global health burden that is set to grow in the coming decades [1, 2]. Alcohol-related liver disease and non-alcoholic fatty liver disease (NAFLD) are two of the most common aetiologies and are precipitated, respectively, by excessive alcohol consumption and the combination of high calorie diet and sedentary lifestyle [3, 4]. The growing prevalence of NAFLD, in particular, parallels that of obesity, type 2 diabetes and other features of metabolic syndrome [5]. The pathology of each of these disorders, as well as chronic infection with hepatitis B (HBV) or hepatitis C (HCV) viruses, manifest inflammatory and pro-fibrogenic processes in the liver that may progress to cirrhosis and hepatocellular carcinoma (HCC). Accordingly, chronic liver disease is a leading cause of mortality in many parts of the world [6, 7].

By way of example, independent of other factors, the average all-cause mortality among NAFLD patients is 11.7% higher compared to individuals without the disease (hazard ratio (HR) 1.93 [1.86–2.00]). The impact of NAFLD on mortality increases with increasing disease severity and ranges from 8.3% (HR 1.71 [1.64–1.79]) for simple steatosis up to 18.4% (HR 2.44 [2.22–2.69]) for non-alcoholic steatohepatitis (NASH) with fibrosis [8]. Adding to the challenge, the capacity to treat NAFLD diminishes with increasing disease severity. Targeted weight loss slows progression in mild disease, but is less effective in moderate to severe disease [9]. Importantly, while there have been considerable breakthroughs in the prevention and treatment of viral hepatitis in recent years [10], no medicine is currently approved for NAFLD and progress has been slow with costly failures in late phase trials due to an inability to easily monitor treatment response.

Despite significant shortcomings in accuracy and practicality, liver biopsy remains the gold standard diagnostic tool to assess the presence and stage of various liver diseases. This technique is currently the most reliable way to determine the pattern and severity of inflammation and fibrosis [11]. For patients with NAFLD, a diagnosis of the more severe form steatohepatitis (NASH) can only be made by histological identification of cardinal features, such as hepatocellular ballooning and lobular inflammation [5]. Since liver biopsy is a highly invasive technique, it comes with the risk of severe complications and cannot be regularly repeated to track changes in the liver over time [3]. Moreover, the technique is associated with considerable interobserver and sampling variability, produces only a limited representation of total liver tissue and, consequently, often underestimates disease severity [12]. These issues limit its widespread and repeated use and give rise to the urgent need for non-invasive biomarkers, to aid diagnosis and monitoring of patients with chronic liver disease. Currently, various scoring systems may be applied to non-invasively stratify patient risk, such as FIB-4 index, Maddrey Discriminant Function (MDF) and Model for End-stage Liver Disease (MELD), which rely on blood biochemistry. Non-invasive diagnoses may employ imaging studies (e.g. magnetic resonance imaging and ultrasound) [13, 14] and liver stiffness may be assessed via transient elastography (e.g. FibroScan) to estimate the degree of fibrosis [15].

In 2019, the American Association for the Study of Liver Diseases identified the insufficient performance of these current non-invasive tools to diagnose early disease and track progression as the critical barrier to treating chronic liver diseases [16]. The limitation being these approaches lack specificity and sensitivity, particularly for mild and early disease. To meet this demand, considerable research effort has focussed on the development of blood-based biomarkers that can reflect early pathological processes, disease progression and response to treatment [11]. In recent times, circulating extracellular vesicles (EVs) have emerged as a potential source of such biomarkers. These nanosized particles contain a distinct molecular signature of protein, RNA and lipid moieties, that is both indicative of their cell type of origin, and also the homeostatic or pathological stimuli that induced their release [17]. EVs are shown to play a role in immune modulation and autoimmune disease, tissue repair, neurodegenerative disease, cardiovascular disease and the development and proliferation of tumours [18]. A breadth of work now evidences the crucial biological activities of EVs in multiple facets of chronic liver pathophysiology, including the cell injury, inflammation and fibrosis shared across diverse aetiologies [5]. Technological developments in high-throughput multi-omics approaches promise to unveil the intricacies of EV molecular cargo and streamline the clinical application of highly sensitive, disease-specific biomarkers [19].

The purpose of this review is to summarise the key works that establish how EVs contribute to normal liver physiology and processes central to the development and progression of chronic liver diseases. The current state and future direction of circulating EV biomarker analyses will also be explored, with a particular focus on techniques to selectively isolate and analyse cell- or tissue-specific EVs for the detection and tracking of chronic liver diseases.

## Extracellular vesicles

EVs are a heterogenous population of small, non-replicating, membrane-encapsulated particles, released by virtually all cell types. Alongside soluble factors and signalling molecules, they have emerged as a fundamental constituent of the cellular secretome [20]. Regular release under basal conditions contributes to the maintenance of homeostasis, while changes to the magnitude and composition of EVs communicate responses to stressful or pathological stimuli between neighbouring and distant cells. Signalling is mediated by receptor-ligand interactions on the EV and cell surfaces, which may directly trigger intracellular pathways or result in the fusion or internalisation of vesicles and their associated cargo [21]. The importance of the role of EVs in intercellular communication is underscored by its evolutionary conservation [11]. Signalling or regulatory molecules transferred in this way are stable and protected from degradation, may be transported through the systemic circulation to distant organs and can easily be taken up by target cells. Notably, the expression of specific surface proteins, such as integrins, promote homing of EVs to target recipient cells [22].

## EV subtypes

As the field of EV research has matured, so too has the complexity of defining distinct EV subpopulations. Vesicles secreted not only by different cell types, but also from the same cell, possess inherent heterogeneity in physical and biochemical properties [19]. Conventionally, EV subtypes are characterised based on their mode of biogenesis. Exosomes, typically 50–150 nm in diameter, are produced via the endosomal pathway. Inward protrusions of the early endosomal membrane create intraluminal vesicles (ILVs) which leads to the formation of multivesicular bodies (MVB). MVB trafficking and fusion to the plasma membrane results in the extracellular release of ILVs, thereby giving rise to exosomes. The production of exosomes may be dependent or independent of the endosomal sorting complex required for transport (ESCRT) machinery. ESCRT-0, -I, -II, and -III protein complexes associate sequentially to facilitate membrane fission and loading of EV cargo [22]. ESCRT-independent exosome release occurs via the production of ceramide and sphingolipid membrane rafts and the activity of neutral sphingomyelinase 2 [23]. Alternatively, microvesicles (MVs), 100–1000 nm in size, shed directly from the plasma membrane. Specific membrane domains are enriched with proteins that permit curvature and budding via higher order oligomerisation and rearrangement of actin-cytoskeletal networks. ESCRT proteins and ceramides are also implicated in MV formation, in addition to ADP-ribosylation factor 6 (ARF6) which participates in cargo selection [22]. MV formation is highly dependent on calcium influx and amenable to activation by cell stress [24, 25].

Given the challenge of identifying the exact intracellular origin of EVs isolated from the extracellular milieu, other characteristics such as size, density and expression of specific surface markers are employed to distinguish EV subpopulations. Though, a recent report of comprehensive EV proteomic characterisation revealed significant heterogeneity in marker expression within subtypes, particularly amongst small EVs with or without endosomal origin [26]. Importantly, most of the commonly used isolation techniques produce a mixture of vesicle populations of varying purity and enrichment. Accordingly, current guidance imparted by the Minimal Information for the Study of Extracellular Vesicles (MISEV) [27], states that isolates should be described generically as “extracellular vesicles”, but may be classified as small EVs (< 200 nm) or medium/large EVs (> 200 nm), by specific molecular components (e.g. ASGR1 + EV) or by cell of origin (hepatocyte-derived EV). It should be noted that for the purpose of biomarker discovery, rigorous separation of EV subtypes may only be necessary to the degree to which sufficient sensitivity and specificity can be achieved.

## EV composition and cargo

**Fig. 1:**
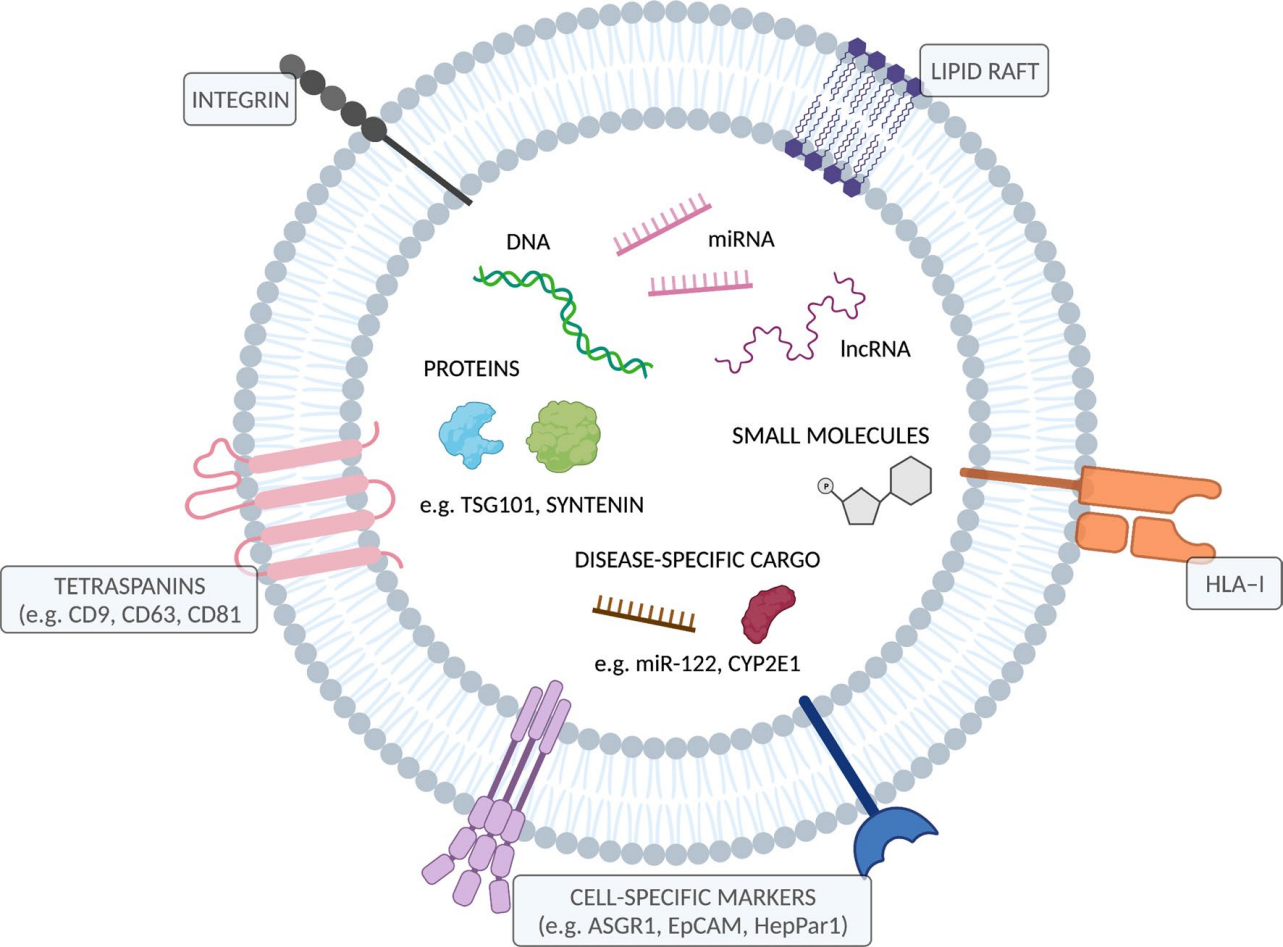
Structure and cargo of an extracellular vesicle. Figure was created using BioRender.com

EVs contain biologically functional cargo, comprised of proteins (including metabolically active enzymes), lipids, metabolites and nucleic acids, such as messenger RNA, microRNA, long non-coding RNA and DNA [28] (Fig. 1). EV-enriched proteins are largely derived from their pathways of biogenesis. Tetraspanins (CD63, CD81 and CD9) and human leukocyte antigen class 1 (HLA-I) are transmembrane proteins commonly found in EV membranes, while tumour susceptibility gene 101 (TSG101), ALG-2 interacting protein (ALIX) and syntenin are cytosolic proteins involved in EV formation that are ultimately exported in vesicles [27]. In addition to general markers of EVs, cell type-specific proteins expressed on cell membranes may be integrated into the membrane of secreted EVs [29]. The identification of cell-type specific surface proteins on EVs has been exploited for immunoaffinity-based isolation of cell- or tissue-specific EVs from the global circulating pool. This has vast potential to improve the sensitivity and specificity of low abundance and ubiquitously expressed disease biomarkers against the background noise resulting from constitutive systemic EV release.

Current evidence for the selective packaging of EV molecular cargo is supported by high variability and discordance in protein and RNA levels between EVs and their parental cell [7, 22]. While the exact mechanisms for regulated sorting of cargo remain unclear, the roles of various RNA-binding proteins, Rab GTPases, and post-translational modifications, such as ubiquitination and phosphorylation, have been reported [24, 30]. The abundance and composition of EVs may be altered in response to ER stress [31] or phenotypic activation. Li, et. al. [7] demonstrated that, compared to quiescent hepatic stellate cells, EV were released at 4.5-fold greater rate upon transdifferentiation to a myofibroblastic phenotype, and contained more abundant proteomic information associated with extracellular matrix production and metabolic activity.

## EVs as minimally invasive biomarkers

EVs are considered attractive biomarkers for a host of reasons. Vesicles are abundant and highly stable in biofluids, exhibiting longer half-lives than other circulating components, such as free proteins or RNA complexes [20]. Durable lipid bilayer membranes protect molecular cargo from degradation, thereby providing a sort of “biomarker reservoir” [32]. Since this diverse cargo is dynamic in nature, directly related to the phenotype of parent cells, it may be used to understand function at the organ, tissue or cellular level and track changes in real time. In line with this application, and in contrast to traditional tissue biopsy, sampling of EVs is easily performed through access to peripheral blood and is repeatable with minimal patient risk. As will be explored throughout this review, the pertinence of EVs as a biomarker source is underpinned by the biological activity of these entities across elements of chronic liver disease. These mechanistic links may be the key to establishing a disease-specific molecular signature from affected tissues. Notably, changes in EVs have been demonstrated at earlier stages than overt tissue damage or other clinical and histological signs [33]. However, as total blood EV is comprised of vesicles released from multiple tissues into the circulation, the development of biomarker strategies is increasingly geared towards selective analysis based on tissue-specific markers [34].

## EV-based therapeutics

In addition to their role as a key diagnostic and monitoring tool for the treatment of liver diseases, the application of EVs as a therapeutic intervention for multiple forms of liver disease has emerged. The properties of EV membranes make them ideal vehicles for therapeutic cargo, including miRNA, small interfering RNA (siRNA), chemotherapy agents or other drugs, which may act to promote tissue regeneration, reduce or reverse inflammation and fibrosis, or target cancer cells in the liver. Promising results have been demonstrated regarding the use of mesenchymal stem cell-derived EVs in various pre-clinical models. However, the requirements to initiate human trials are very different between a biomarker and an intervention. EV-based therapeutics face several challenges related to the cost and scale of manufacturing pure EVs that adhere to regulatory and quality control standards for use in humans. Meanwhile, much of the recent research regarding the role of EVs as biomarkers has come from human data. Beyond pre-clinical studies identifying EV cargo that reflect molecular changes in liver diseases, a key focus of the present review is the detection of circulating EVs in human patients. Thus, the application of EVs as therapeutics will not be extensively reviewed here but may be found in references [22, 35, 36].

## EVs in normal liver physiology

**Fig. 2:**
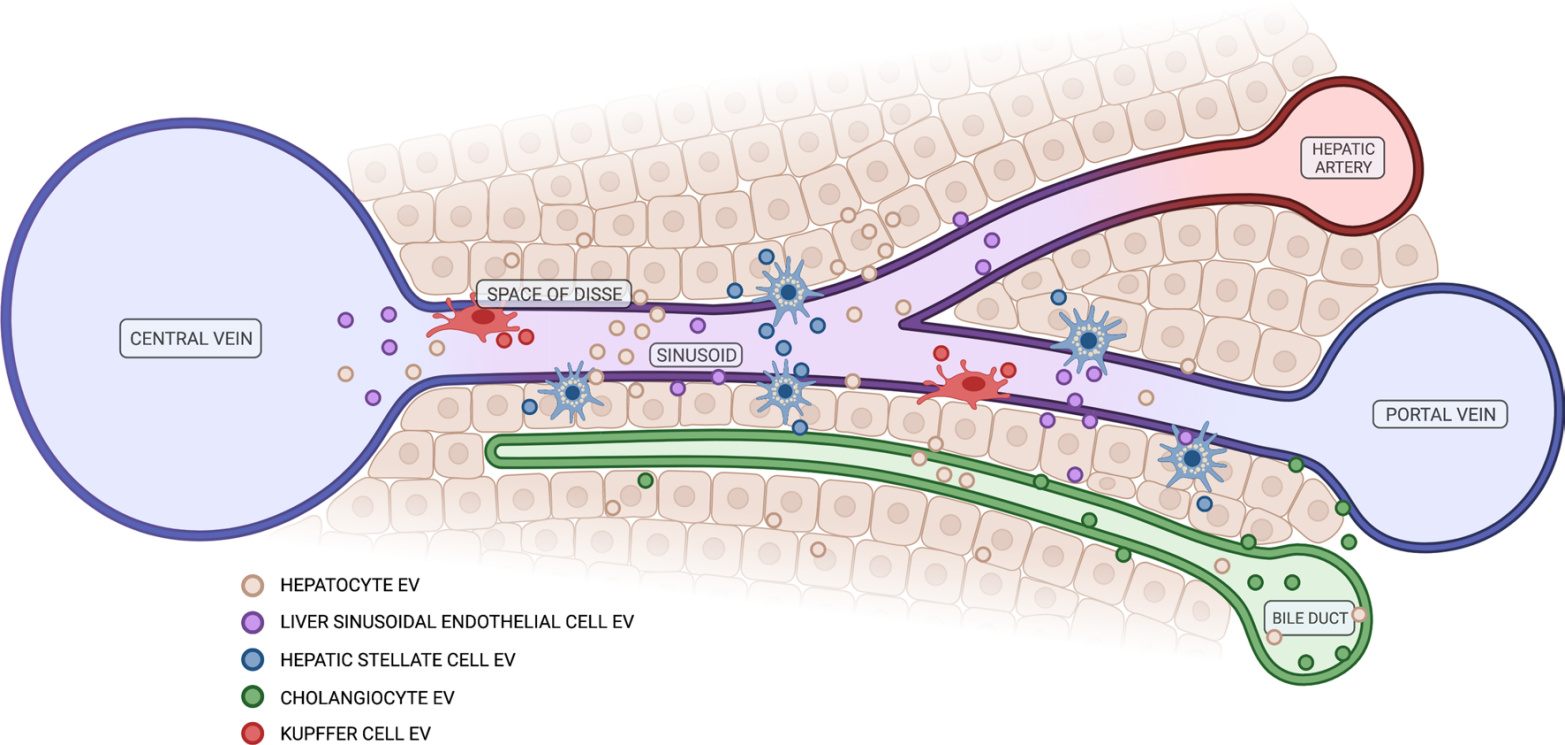
Extracellular vesicle release by various hepatic cells in normal liver function. Figure was created using BioRender.com

The liver is the largest internal organ in the body, functionally and anatomically complex and responsible for a diverse set of metabolic, synthetic, digestive, detoxifying, storage and regulatory roles. Approximately, 80% of total liver volume is comprised of hepatocytes, which are responsible for the central physiological processes, while a further 6.5% accounts for non-parenchymal cells that function in support of hepatocytes and maintenance of the hepatic microenvironment [20, 37]. These cells include liver sinusoidal endothelial cells (LSECs), hepatic stellate cells (HSCs), cholangiocytes and the population of liver-resident macrophages, known as Kupffer cells. The organised lobular architecture of the liver facilitates cooperation and inter-regulatory functions of diverse cell types through anatomical proximity [38]. Effective cell-to-cell communication is also achieved by the network of EV interactions, as each cell is both a donor and recipient of EVs from the same and other hepatic cell types (Fig. 2). The bi-directional transfer of molecular information is imperative to homeostatic control in the liver as well as the broader inter-organ communicative landscape.

## Hepatic cell-derived EVs

The function of EVs derived from different hepatic cell types is summarised in Table 1. Multiple enzymes involved in the metabolism of carbohydrates, lipids, endogenous compounds and xenobiotics are among key molecular cargo identified in hepatocyte-derived EVs [39]. Hepatic metabolic activity may be transferred to or induced in extra-hepatic niches. For example, hepatocyte-derived EVs carrying arginase-1 were found to regulate endothelial cell function and alter serum metabolites associated with oxidative stress in the systemic vasculature [40]. In the liver, hepatocyte-EVs have also been shown to promote the proliferation of cholangiocytes and other hepatocytes in paracrine and autocrine fashions, respectively [37, 41]. Hepatocyte-derived EVs have demonstrated the remarkable capacity to mediate regeneration of functional liver mass. Nojima, et. al. [42] EV-mediated transfer of sphingosine-1-phosphate (S1P), sphingosine kinase 2 (SK2) and ceramidase between hepatocytes promoted liver regeneration in mice following 70% hepatectomy. HSC are the key fibrogenic cells in the liver, and exchange of EVs between them is crucial in balancing extracellular matrix (ECM) production and degradation. LSEC-derived EVs contribute to modulation of this balance. The EVs normally maintain HSC quiescence, but when stimulated, EVs containing upregulated sphingosine kinase 1 (SK1) are released to activate HSC [43]. Quiescent HSC (qHSC) release EVs containing miRNAs (miR-214 and -199-5p) and the transcription factor, Twist-1 [44, 45]. This cargo suppresses connective tissue growth factor (CTGF) to maintain quiescence in other qHSC or downregulate pro-fibrotic genes, including α-smooth muscle actin (αSMA) and collagen, in activated HSC (aHSC) [7, 21]. Conversely, aHSC-derived EVs promote ECM production by transferring CTGF [46]. Lastly, EVs from cholangiocytes participate in bile acid homeostasis through the transfer of long non-coding RNA H19 to hepatocytes [47], and in wound-healing responses by delivering hedgehog ligands to promote angiogenesis, growth and differentiation in recipient LSECs [48].

**Table 1:** Hepatic cell EVs function and cargo

Originating cell	Example cargo	Recipient cells	Functions	References
Hepatocytes	DMET proteins and mRNA	Hepatocytes, extrahepatic cells	Transfer metabolic activity	Conde-Vancells et al. [39], Kumar et al. [51], Rowland et al. [52], Rodriguez et al. [53]
	Arginase-1	Endothelial cells	Regulate endothelial cells in systemic vasculature	Royo et al. [40]
	S1P, SK2, ceramidase	Hepatocytes	Promote proliferation and liver regeneration	Nojima et al. [42]
Liver sinusoidal endothelial cells	SK1	HSC	Modulate quiescent/active phenotype	Wang et al. [43]
Hepatic stellate cells	miR-214, miR-199-5p, Twist-1, CTGF	HSC	Modulate quiescent/active phenotype	Charrier et al. [46], Chen et al. [21], Chen et al. [45], Chen et al. [44] and Li et al. 2020
Cholangiocytes	lncRNA H19	Hepatocytes	Regulate bile acid homeostasis	Li et al. [47]
	Hedgehog ligands	LSEC	Promote wound-healing response	Witek et al. [48]

## Metabolism

Hepatic metabolism plays a critical role in regulating the abundance of endogenous chemicals, such as bile acids, fatty acids, steroid hormones and bilirubin. Similarly, it serves as a major clearance mechanism for xenobiotics including drugs, dietary chemicals and environmental toxins. Specifically, metabolic clearance is the major route of elimination for more than 80% of pharmaceutical drugs [49, 50]. Notably, the mRNA transcripts and active proteins of drug metabolising enzymes and transporters (DMET), cytochrome P450 (CYPs), UDP-glucuronosyltransferase (UGTs), glutathione S-transferase and organic anion transporting polypeptides (OATPs) have been detected in EVs derived from hepatocytes and in the blood. CYP protein and mRNA is enriched in circulating EVs relative to total plasma, which suggests selective packaging [51–53]. The transfer of DMET in circulating EVs has physiological significance with respect to protection of extra-hepatic cells from systemic toxicants or increasing metabolic activity in tissues with lower basal DMET expression, such as the lungs or brain [54]. Clinically, this notion has potential applications as liquid biopsy to indicate chronic alcohol, nicotine or illicit drug use, liver disease, or to assess metabolic drug–drug interactions (DDIs) and inter-individual variability in drug exposure. For example, CYP2E1 is induced by chronic use of alcohol or paracetamol overdose. This is reflected in greater release of hepatocyte EVs that transfer the capacity for CYP2E1-mediated metabolism, resulting in oxidative stress and acute injury in hepatic and non-hepatic cells [51]. A recent review described how disease-associated alterations in CYP protein expression and activity may impact drug exposure in patients with NAFLD [55]. The capacity to monitor changing pharmacokinetic profile is paramount for the development of novel therapeutics for NAFLD and in optimal dosing of existing treatments for common comorbidities.

Assessing variability in metabolic clearance within or between individuals, resulting from variable hepatic DMET expression or activity, DDIs, presence of liver disease or other factors, is an appealing avenue for EV-based DMET profiling. Work by our group showed that EV-derived CYP3A4 was highly concordant with apparent oral clearance of its probe substrate midazolam in healthy subjects pre- and post-dosing of the inducer, rifampicin [52]. Since then, Achour, et. al. [49] evaluated hepatic elimination based on circulating EV mRNA of clinically important DMET, reporting sound correlations with protein expression in liver tissue. Interestingly, this study normalised the data to a panel of 13 liver-specific RNA markers (e.g. apolipoprotein A2 and fibrinogen-beta) as part of a novel shedding factor to account for variability in liver EV release into the bloodstream. Instead, we recently applied our novel two-step anti-ASGR1 immunocapture technique to selectively isolate hepatocyte-derived EVs from global EVs and successfully tracked the induction of CYPs 3A4, 3A5 and 2D6 and OATPs 1B1 and 1B3 during pregnancy and following rifampicin administration [53]. Together these reports position EV liquid biopsy as a viable strategy for individual DMET phenotyping to aid precision dosing or classification of clinical trial participants at enrolment.

## EVs in liver pathobiology

Chronic liver diseases result from prolonged injurious stimuli that exceed the regenerative capacity of the liver. Over time, unresolved inflammatory and fibrogenic activation from disorders such as ALD, NAFLD, HBV and HCV infection can ultimately lead to fibrosis, cirrhosis and HCC [20]. EVs have emerged as potent pathogenic drivers in several of these processes and a breadth of pre-clinical data establishes the key molecular information carried in EVs that mediate liver cell cross-talk in different chronic liver diseases. In several instances, these EV cargoes have been analysed in the circulation of animal models or human patients and demonstrate the capacity for circulating EVs derived from specific cellular sources to reflect pathological events in affected organs. For each chronic liver disease, Table 2 lists the cell-specific EVs and their cargoes, with recipient cells and resulting function (if defined), divided into studies that examined EVs in circulation and in vitro studies of EV cargo yet to be translated to circulating EVs.

**Table 2:** EV cargo from specific cellular origins, effect on recipient cells and detection in circulation in chronic liver diseases

Condition	Originating cell (or source)	Cargo	Recipient cells	Function	Additional information	References
NAFLD	*Cargo detected in circulating EVs*					
	Hepatocytes	miR-122, -192, -128-3p	N/A	N/A	Increased in plasma of NAFLD patients	Newman et al. [68]
	Hepatocytes	mtDNA	N/A	TLR9 activation → inflammation	Increased in plasma of NASH patients	Garcia-Martinez et al. [70]
	Hepatocytes	ASGR1, CYP2E1	N/A	N/A	Increased in plasma of mice over-expressing IRE1α	Dasgupta et al. [31]
	Adipocytes	miR-99b	Hepatocytes	Suppress FGF-21 → promotes steatosis	Demonstrated transfer of cargo via circulation in mice with genetically altered miRNA processing in adipocytes	Thomou et al. [72]
	Hepatocytes Macrophages Neutrophils Platelets	ASGR1, CYP2E1 Galectin 3 Ly-6G/6C CD61	N/A	N/A	Increased in NAFLD mouse model	Li et al. [33]
	Hepatocytes	ASGR1, SLC27A5	N/A	N/A	Increased in NASH patient serum	Povero et al. [3]
	NK T-cells Macrophages	Valpha24/Vbeta11 CD14	N/A	Inflammation	Increased in NAFLD patient serum	Kornek et al. [85]
	Hepatocytes	ASGR2, CYP2E1	N/A	N/A	Decreased in patients with NAFLD resolution	Nakao et al. [109]
	Hepatocytes	ASGR1, HepPar1	N/A	N/A	Decreased in patients with bariatric surgery	Rega-Kaun et al. [131]
	*Cargo not yet analysed in circulating EVs*					
	Lipotoxic hepatocytes	TRAIL	Hepatocytes, macrophages	Hepatocyte death, macrophage activation & pro-inflammatory cytokine (IL-1β, IL-6)	Upregulated, in vitro	Hirsova et al. [62]
	Lipotoxic hepatocytes	CXCL10	Macrophages	Macrophage chemotaxis	Upregulated, in vitro	Ibrahim et al. [63, 64]
	Lipotoxic hepatocytes	C16:0 ceramide, SK1	Macrophages	Macrophage chemotaxis	Upregulated, in vitro	Kakazu et al., [65], Dasgupta et al. [31]
	Lipotoxic hepatocytes	miR-128-3p	HSC	Suppress PPARγ → profibrotic gene expression	Upregulated, in vitro	Povero et al. [66]
	Lipotoxic hepatocytes	VNN1	LSEC	Promote pathologic angiogenesis	Upregulated, in vitro	Povero et al. [67]
	Adipocytes	MCP-1, IL-6, MIF	Hepatocytes	Promote insulin resistance	Adipose tissue explant EVs applied to hepatocytes in vitro	Kranendonk et al., [71]
	Adipocytes	miRNA profile	Hepatocytes, HSC	Target TGF-β pathway → inhibits fibrolytic enzymes e.g. MMP-7	Adipose tissue EVs applied to hepatocytes in vitro	Koeck et al., [18]
ALD	*Cargo detected in circulating EVs*					
	Hepatocytes	mtDNA	Macrophages	TLR9 activation → pro-inflammatory cytokine release (IL-1β, IL-17)	Increased in murine AH model	Eguchi et al. [75]
	Hepatocytes	mtDNA	Neutrophils	Neutrophilia, macrophage recruitment to liver	Increased in murine chronic-plus-binge ethanol feeding model	Ma et al., [76]
	Hepatocytes	CD40 ligand	Macrophages	Phenotypic activation → Upregulated pro-inflammatory cytokine (IL-1β, IL-6, TNF-⍺)	Increased in human AH patients	Verma et al., [77]
	Hepatocytes	miR-122	Monocytes	Suppress haem oxygenase 1 → sensitise to pro-inflammatory stimuli	Increased in human acute alcohol use and mice binge and chronic alcohol consumption	Momen-Heravi et al., [78]
	Serum EV	miR -122, -155	N/A	N/A	Increased in EV-fraction of circulation in mice	Bala et al., [79]
	Serum/plasma EV	let-7f, miR -29a, -340	N/A	Target genes involved in inflammation and cancer development	Increased in mice with AH	Eguchi et al., [17]
	Hepatocytes	ASGR2, CYP2E1 Sphingolipids	N/A	Promote inflammation and cell death in AH	Increased in AH patient serum	Sehrawat et al., [13]
Viral Hepatitis	*Cargo detected in circulating EVs*					
	HCV-infected hepatocytes	Replication competent HCV-RNA	Hepatocytes	Viral transmission	Identified in human HCV patients	Bukong et al., [83]
	HCV-infected hepatocytes	miR-19a	HSC	TGF-β pathway activation → profibrotic gene expression	Upregulated in human HCV patients	Devhare et al., [84]
	CD8 + and CD4 + T cell	CD147	HSC	Induce MMP enzymes → promote ECM degradation in HCV-related fibrosis	Increased in active HCV patients	Kornek et al., [85]
	*Cargo not yet analysed in circulating EVs*					
	HBV-infected hepatocytes	HBV RNA and protein	Peripheral blood monocytes	Induce PDL-1 expression	Identified in vitro	Kakizaki et al., [81]
	HCV-infected hepatocytes	HCV protein E2	N/A	Mimic viral particles → hinders neutralising antibody response	Identified in vitro	Deng et al., [82]
	LSEC	IFN-stimulated genes	Hepatocytes, LSEC	Promote antiviral response	Identified in vitro	Giugliano et al., [38]
HCC	*Cargo detected in circulating EVs*					
	Malignant hepatocytes	miR-93 miR-224 miR-665	Hepatocytes	Promote HCC proliferation	Each upregulated in human HCC patients	Xue et al., [89], Cui et al., [90], Qu et al., [91]
	Malignant hepatocytes	miR-9-3p miR-638 miR-718 miR-744	Hepatocytes	Inhibit HCC proliferation	Each downregulated in HCC patients	Tang et al., [92], Shi et al., [93], Sugimachi et al., [94], Wang et al., [95]
	Malignant hepatocytes	miR-1247-3p	Fibroblasts	Phenotypic switch to cancer-associated fibroblasts in lung metastasis, increased pro-inflammatory cytokine (IL-6 and IL-8) secretion	Increased in HCC patients with lung metastasis	Fang et al., [99]
	Tumour (HCC/ICC/PSC) EV in serum	Proteomic signature	N/A	N/A	Differential expression between pathologies and healthy controls	Arbelaiz et al. [100]
	Tumour (HCC) EV in serum	Annexin V, EpCAM, ASGR1, CD133	N/A	N/A	Panel of markers distinguishes HCC from cholangiocarcinoma	Julich-Hartel et al., [125]
	Tumour (HCC) EV in plasma	HepPar1	N/A	N/A	Increased with HCC recurrence	Abbate et al. [126]
	Tumour (HCC) EV in plasma	ASGR1, EpCAM, CD147, 10 mRNA transcripts	N/A	N/A	Differential expression between HCC, other primary malignancies and non-cancer	Sun et al. [32]
	*Cargo not yet analysed in circulating EVs*					
	Malignant hepatocytes	miRNA profile	Hepatocytes	Modulate TAK1 pathway → promote cancer growth	Identified in vitro	Kogure et al. [96]
	Malignant hepatocytes	linc-ROR	Healthy hepatocytes	Inhibit apoptosis and enhance proliferation	Upregulated in vitro	He et al. [97]
	Malignant hepatocytes	MET proto-oncogene, caveolins, S100 family members	Healthy hepatocytes	Mobilisation, tumour invasion	Identified in vitro	He et al. [98]
Fibrosis	*Cargo detected in circulating EVs*					
	Activated HSC	PDGFR⍺	HSC	Promote migration	Upregulated in human liver fibrosis patients	Kostallari et al. [30]
	LSEC	SK1, S1P mRNA and protein	HSC	Promote AKT phosphorylation and migration	Upregulated in human AH patients and mice with experimental liver fibrosis	Wang et al. [43]
	Hepatocytes, activated HSC	Hedgehog ligands	HSC, endothelial progenitor cells	Promote proliferation and angiogenesis	Upregulated in rats undergoing bile duct ligation	Witek et al. [48]
	Serum EV	miR -34c, -151-3p, -483-5p, -532-5p and -687	Hepatocytes, activated HSC	Decrease hepatocyte death, hepatic fibrosis and inflammation	Downregulated in human liver fibrosis patient and mice with experimental liver fibrosis	Chen et al. [1]
	*Cargo not yet analysed in circulating EVs*					
	Activated HSC	Proteomic profile associated with ECM production and metabolic activity	HSC	Activate HSC, promote fibrogenesis	Identified in vitro	Li et al. [7]

## Non-alcoholic fatty liver disease

NAFLD is the most common chronic liver disease, currently estimated to affect more than 25% of the global population [56]. The condition may be considered a hepatic manifestation of the metabolic syndrome as it is often implicated with other features, such as insulin resistance (IR), obesity and type 2 diabetes mellitus [5]. In line with this, recent expert consensus supports the updated nomenclature of metabolic associated fatty liver disease (MAFLD), to reflect advancing knowledge of disease phenotype, heterogeneity in drivers and coexisting conditions and diagnostic criteria that is based on inclusion rather than exclusion (particularly around alcohol use) [57, 58].

The condition presents as a spectrum of clinical disease with some patients exhibiting simple steatosis (NAFL) while a fraction (~ 30%) will develop non-alcoholic steatohepatitis (NASH) [59]. NAFLD is the product of multiple dysregulated signalling pathways in the liver that involves abnormal lipid metabolism leading to lipotoxicity and inflammation [31]. While several risk factors relating to diet and lifestyle are linked to the incidence of NAFLD, genetic predispositions have also been noted, as recently reviewed by Jonas, et. al. [60]. Further, the contribution of gut dysbiosis, liver-adipose cross-talk and increased cardiovascular disease-related mortality, underscores the systemic nature of this condition [61]. Current diagnostic tools remain inadequate for the early detection, risk stratification and monitoring of NAFL and NASH, presenting a significant hindrance to the clinical management of patients and development of effective pharmaceutical interventions [16].

Numerous reports to date demonstrate changes in EVs released by hepatocytes under lipotoxic stress and their contributions to cellular and inter-organ cross-talk to promote inflammation and fibrosis in the liver. These were described in detail in a previous review [5]. Key molecular cargo of lipotoxic hepatocyte-derived EVs include the death receptor ligand, TRAIL, which triggers hepatocyte death and macrophage activation with increased pro-inflammatory cytokine expression (interleukin [IL]-1β and IL-6) [62]. The macrophage chemoattractant C-X-C motif ligand 10 (CXCL10) was also detected in EVs induced by steatosis-related JNK activation in the liver [63, 64]. Further, lipotoxic EV release was found to be dependent on ceramide pathways, activated by the ER stress sensor inositol-requiring enzyme-1α (IRE1α). IRE1α-stimulated EVs contained C16:0 ceramide and SK1 which promoted macrophage chemotaxis in vitro [65] and macrophage recruitment and hepatic inflammation in mice [31]. The authors also showed that mice over-expressing IRE1α had significantly elevated circulating EVs and their hepatocellular origin was identified by electron microscopy (EM) with immunogold labelling of ASGR1 and CYP2E1. Lipotoxic hepatocyte-derived EVs also modulate HSC phenotype in NAFLD. Specifically, EVs containing miR-128-3p suppressed peroxisome proliferator-activated receptor-γ (PPARγ) in HSC, resulting in upregulated profibrotic gene expression [66]. The effect was dependent on EV internalisation by HSC, mediated by Vanin-1 (VNN1) on the surface of vesicles. Increased VNN1 expression on lipotoxic EVs was previously implicated with EV internalisation by LSEC resulting in pathologic angiogenesis [67]. Increased expression of miR-128-3p was also identified in our recent work, alongside miR-122 and -192, in NAFL and NASH patient plasma EVs. This was only observed in circulating EVs derived specifically from hepatocytes (expressing ASGR1) [68]. Mitochondrial dysfunction and oxidative stress are common pathogenic events in fatty liver diseases related to both aetiologies, non-alcoholic and alcoholic (discussed in the following section) [69]. Mitochondrial DNA (mtDNA) has been identified as important EV cargo that promotes inflammation via TLR9 activation, thereby contributing to the transition from simple steatosis to steatohepatitis. Garcia-Martinez, et. al. [70] found greater levels of mtDNA in plasma microvesicles of mice and patients with NASH, with concomitant increase in hepatocyte-specific marker, Arg-1, and demonstrated the capacity for these particles to activate TLR9.

EVs from visceral adipose tissue actively contribute to NAFLD pathogenesis by exacerbating systemic IR, inflammation and hepatic fibrosis [71]. Differentially expressed miRNAs in adipocyte-EVs from lean and obese individuals target the TGF-β pathway in hepatocytes and HSC, resulting in the inhibition of fibrolytic enzymes such as matrix metalloproteinase (MMP)-7 [18]. Another study emphasised the important contribution of adipocyte-EV to circulating miRNA levels and their capacity to modulate gene expression in the liver [72]. The authors showed that fibroblast growth factor (FGF)-21 is a liver protein target of adipocyte-EV derived miR-99b. FGF-21 is implicated in many metabolic pathways and its suppression contributes to hepatic steatosis [73]. In all, the current evidence positions EVs as key players in the pathogenesis and progression of NAFLD and supports the investigation of biomarkers within EV derived from adipocytes and hepatic cell populations.

## Alcoholic liver disease

Alcoholic liver disease (ALD) follows a similar clinical course to that of NAFLD. Hepatic steatosis and alcoholic hepatitis (AH) may resolve with alcohol abstinence, but progressive disease can lead to cirrhosis and liver failure [74]. Liver biopsy is not usually necessary for ALD diagnosis as a history of significant alcohol consumption along with clinical, radiologic and biochemical findings are often sufficient. However, diagnosis may be complicated in alcoholic patients with unreliable history or co-existing risk factors for other conditions such as NAFLD; in such cases the threshold for “significant” alcohol intake may be reduced. The lack of accurate non-invasive biomarkers limits the dynamic assessment of inflammatory activity and degree of fibrosis in ALD, as well as the risk of developing cirrhosis. Considering that ALD accounts for 50% of cirrhosis-related deaths [13], biomarker discovery is an area of intense research focus to improve the management of ALD and development of pharmacological strategies to halt or reverse the disease.

EV-mediated macrophage activation is increasingly recognised as a key feature of the inflammatory process in AH and parallels hepatic injury and fibrosis. A mouse model of AH had significantly increased EV levels in circulation and vesicles isolated from primary hepatocytes were found to be enriched in mtDNA [75]. These EV activated TLR9 resulting in upregulated pro-inflammatory cytokine, IL-1β and IL-17 production in liver macrophages and promoted fibrogenic activation of HSC. Another study similarly found that hepatocyte-derived mtDNA-enriched vesicles released in response to chronic and binge ethanol feeding in mice contributed to macrophage and neutrophil infiltration in the liver [76]. Verma, et. al. [77] treated hepatocytes with ethanol in vitro, and showed greater release of EVs expressing CD40-ligand. These stimulated macrophage-to-M1 phenotypic switching, characterised by upregulated pro-inflammatory cytokine expression (TNFα, IL-1β and IL-6). Increased CD40L-expressing EVs were also detected in the serum of patients with AH. Similarly, Momen-Heravi, et. al. [78] demonstrated that EV containing miR-122 is transferred from ethanol-treated hepatocytes to monocytes, resulting in suppression of haem oxygenase 1 (HO-1) and subsequent sensitisation to pro-inflammatory stimuli, such as lipopolysaccharide. In addition, mice and humans subjected to acute alcohol binge, and mice also to chronic consumption, had more EVs in circulation. The levels of miR-122 and -155 in the EVs changed over time post-binge, suggesting variable packaging in response to alcohol. This notion is supported by earlier data comparing specific miRNA expression in circulating vesicle or protein fractions across models of liver injury with different aetiologies [79]. The authors showed that in models of inflammatory (i.e. NAFLD) or alcohol-induced disease, miR-122 and -155 was mostly EV-associated, while predominantly protein-associated in DILI. This distinctive distribution of miRNA in chronic liver disease in contrast to the acute condition, DILI, supports the investigation of biomarkers localised in EVs in the circulation to improve performance and disease-specificity. EV miRNA profile was also explored in mice with AH induced by continuous intragastric ethanol infusion. Three miRNAs in blood EVs, including let-7f-5p, miR-29a-3p and miR-340-5p, discriminated AH mice from controls, as well as from obese mice and those with NASH or cholestatic injury [17]. Various sphingolipids have also been implicated with inflammation and cell death in AH. Serum EVs from AH patients were recently shown to be significantly enriched in six sphingolipid species compared to healthy controls, heavy drinkers, NASH patients and alcoholic cirrhosis patients. The cargo was positively correlated with disease severity and predicted 90-day survival [13].

## Viral hepatitis

Viral infections represent a significant cause of chronic liver diseases and are the most common aetiology for HCC [6]. In addition to certain viral factors, the carcinogenic nature of HBV and HCV are linked to chronic inflammation, fibrosis and changes in signalling pathways implicated in hepatocyte survival and tumour surveillance and suppression [80]. While viral infections can be diagnosed by serological techniques and monitored with respect to viral load and immune status [11], a better understanding of the role of EVs in pathogenesis and disease progression may facilitate non-invasive assessment of liver damage and identify early markers of increased HCC risk.

EVs are potent modulators of immune function. Hepatocytes infected with replicating HBV release EVs that induce programmed death ligand-1 (PDL-1) in recipient monocytes, possibly suppressing host antiviral activity [81]. Another study showed that HCV-infected hepatocytes secrete EVs coated with the HCV protein E2. These EV mimic viral particles thereby hindering the neutralising antibody response [82]. Conversely, LSEC-derived EVs stimulated by interferon (IFN)-I and -III, contribute to the antiviral response [38]. Interestingly, EVs were found to participate in viral spread during HCV infection. Using a rigorous multi-step approach to remove free virus contamination, Bukong, et. al. [83] isolated EVs from infected patient sera and Huh7.5 cell culture supernatant. The EVs contained replication competent HCV-RNA in complex with argonaute-2, heat shock protein 90 and miR-122, which mediated new infection in hepatocytes. Hepatocyte-derived EV also activates HSC to promote fibrosis in HCV. TGF-β pathway activation was found to be triggered by EV-derived miR-19a in vitro and increased levels of the miRNA were detected in serum EV from chronic HCV patients compared to healthy controls and patients with non-HCV-related liver disease of similar fibrosis grade [84]. A previous study, however, found elevated circulating CD8 + and CD4 + T cell-derived EVs in patients with active HCV, that promoted ECM degradation by induction of MMP enzymes in HSC [85]. In summation, these reports provide avenues for development of novel biomarkers or therapeutic tools for chronic viral hepatitis.

## Hepatocellular carcinoma

Hepatocellular carcinoma accounts for more than 80% of primary liver malignancies and a third of global cancer-related deaths [86]. Chronic liver diseases, especially with cirrhosis, are major risk factors for HCC. Prognosis is poor, exhibiting only 20% 5-year overall survival, often due to late stage diagnoses [86]. Ultrasound has acceptable sensitivity and specificity for HCC screening, but its capacity to detect early lesions is limited [32]. Serum alpha-fetoprotein (AFP) is a biomarker of widely variable performance that may be elevated in late stages, but only in a subset of patients [11]. Accordingly, a combination of ultrasound and AFP assessment is recommended for surveillance by the Australian practice guidelines [87]. The use of ultrasound is also endorsed by the American Association for the Study of Liver Diseases (AASLD), with or without AFP [88]. EVs are one of three liquid biopsy approaches in oncology, among circulating tumour DNA and tumour cells [32]. Since EVs carrying tumour-derived information are present in circulation earlier and persist through to advanced disease, they present the opportunity to initiate curative interventions.

The dysregulation of multiple signalling pathways and complex network of interactions between malignant and non-malignant cells in the tumour microenvironment are critical to tumour progression. EVs are known to play a role in regulating cell proliferation, migration, angiogenesis, metastasis, epithelial-to-mesenchymal transition (EMT), and immune escape. Specific HCC-EV miRNA cargo, for example, has been linked to enhanced HCC proliferation (including miR-93, -224 and -665) [89–91], while other cargo was found to have an inhibitory effect (miR-9-3p, -638, -718 and -744) [92–95]. Kogure et al. [96] reported that selectively packaged miRNA and protein in HCC-derived EVs modulate the TGF-β activated kinase 1 (TAK1) pathway in other hepatic cells to promote cancer growth. More recently, HCC-derived EVs were found to inhibit apoptosis and enhance proliferation of hepatocytes via transfer of long intergenic non-coding RNA regulator of reprogramming (linc-ROR) [97].

EVs also promote the invasion of HCC tumours through normal liver tissue, as metastatic HCC-derived EV mobilise healthy hepatocytes via transfer of oncogenic cargo, such as mesenchymal-epithelial transition (MET) proto-oncogene, caveolins and S100 family members [98]. EV from metastatic HCC also contain miR-1247-3p which facilitates the conversion of normal fibroblasts to cancer-associated fibroblasts (CAFs) in lung metastases and increased pro-inflammatory cytokine (IL-6 and IL-8) secretion [99]. In HCC patients, lung metastasis was positively correlated with serum levels of EV-derived miR-1247-3p. EV protein cargo in serum was also shown to aid differential diagnosis of intrahepatic cholangiocarcinoma, HCC and primary sclerosing cholangitis, which is challenging with current non-invasive tools [100]. These studies support the role of tumour-derived EV cargo in encouraging a tumour-favourable environment for progression and metastasis through communication with cancerous and non-cancerous cells, and advance the notion that promising biomarker candidates linked to oncogenic processes may be detected in circulating EVs.

## Fibrosis

Liver fibrosis is a significant cause of morbidity and strong independent risk factor for mortality in chronic liver diseases, especially NAFLD [101]. While effective treatment of the precipitating condition may reverse fibrosis in some patients, specific antifibrotic treatment options are scarce [1] and patients may advance to cirrhosis and liver failure, often necessitating liver transplantation [43]. HSC activation is the principal event at the cellular level leading to ECM deposition and, under persistent profibrogenic conditions, can produce fibrous scar and severely compromise liver function [1, 7]. Fibrosis is a typical progression common among multiple chronic liver diseases and can be characterised by a number of molecular pathways not specifically altered by a particular condition. These may be monitored via EV-derived markers as described below; thus, in conjunction with disease-specific markers that identify the precipitating condition, fibrosis markers may be helpful in tracking the severity of this complication.

EVs from HSCs of both quiescent and myofibroblast phenotypes form a complex interplay of pro- and anti-fibrotic EV signalling in the injured liver. One study determined that HSCs treated with PDGF-BB in vitro released EVs enriched with PDGF receptor-alpha (PDGFRα) via a mechanism of selective packaging [30]. The EVs promoted migration in recipient HSC and liver fibrosis in healthy mice, while inhibiting EV export of PDGFRα ameliorated fibrosis in carbon tetrachloride (CCL_4_)-treated mice. Patients with liver fibrosis also had increased levels of PDGFRα in serum EV. HSC phenotype is further modulated by LSEC-derived EV. Wang et al. [43] showed that the EV specifically transfer SK1 and S1P cargo, which upregulate AKT phosphorylation and migration. Expression of each at RNA and protein level was detectable in EVs from mice with experimental liver fibrosis and human patients with alcoholic fibrosis.

The Hedgehog (Hh) pathway is critical to the wound-healing response and tissue remodelling in chronic liver injury. Hh ligands released in EVs from damaged hepatocytes and aHSC, promote proliferation and angiogenesis in recipient HSC and endothelial progenitor cells, respectively; and have been detected at increased levels in the plasma and bile of rats with fibrosis induced by bile duct ligation [48]. Further, discordant miRNA and protein cargo in EVs from qHSC and aHSC, either stimulate or inhibit fibrosis depending on the phenotype of originating and recipient cells. Serum EVs from healthy individuals contain “anti-fibrotic” miRNA which decreased CTGF, αSMA and collagen gene expression when applied to aHSC in vitro and reduced hepatic fibrosis and inflammation in CCL_4_-treated mice [1]. Meanwhile, a proteomic comparison of qHSC- and aHSC-derived EV revealed greater protein content in the latter, associated with profibrotic, inflammatory and chemotactic functions [7]. Accordingly, the presence of distinct pro- and anti-fibrotic EV populations in the liver presents the intriguing possibility to track fibrogenic activity and develop novel antifibrotic therapies.

## Analysis of circulating tissue-specific EV biomarkers

**Fig. 3:**
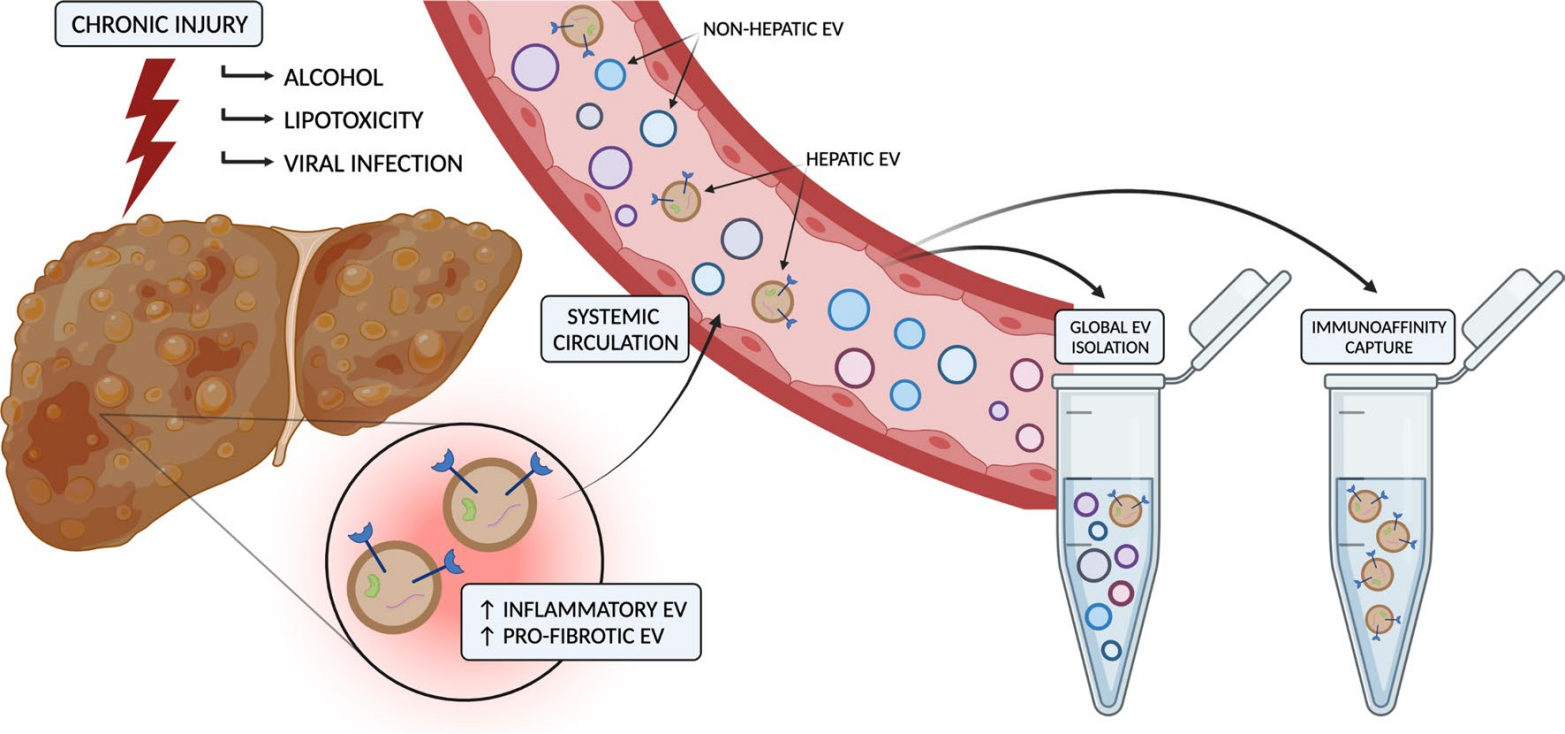
Extracellular vesicle liquid biopsy for chronic liver diseases. Figure was created using BioRender.com

**Fig. 4:**
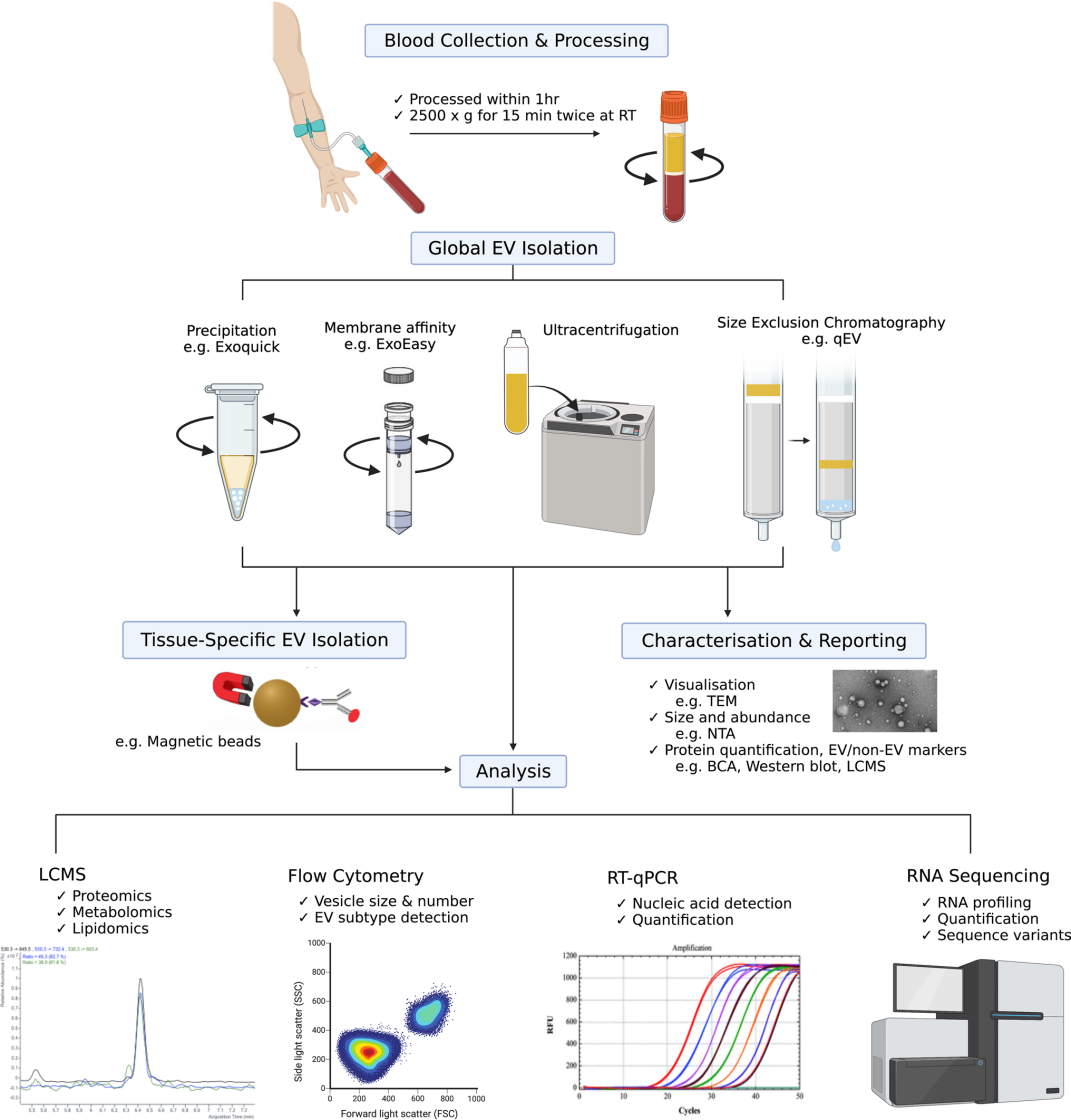
Generic workflow for the sample collection and analysis of extracellular vesicle-derived biomarkers. Figure was created using BioRender.com

The studies described thus far indicate the potential for biomarkers with mechanistic links to chronic liver pathology to be released in EVs and detected in the circulation (Fig. 3). However, the EVs harbouring these molecules of interest account for a relatively small proportion of the complex circulating mixture of vesicles, which are also derived from multiple other cells and organs. In plasma, platelets are a major source of EVs (originating up to 90%), followed by other haematopoietic or endothelial cell types [102, 103]. While the reported proportion of hepatocyte-derived EVs in circulation varies widely [3, 33], this may be as small as a fraction of a percent. Since conventional methods separate vesicles from other blood components based on physical properties, producing bulk isolates of heterogenous composition, the background noise from non-hepatic EVs may preclude the sensitive detection of disease-related changes. This is likely to be particularly apparent in the diagnosis of early-stage patients [104]. Evidently, the inability to efficiently isolate relevant subpopulations of EVs containing candidate biomarkers represents a major barrier to their clinical translation. Immunoaffinity capture fits within a broader framework of EV sample collection and analysis for blood-based biomarker contexts. The methodology can be summarised in a generic workflow (Fig. 4) that incorporates best practice (as reviewed by Useckaite, et. al. [105]) and recommendations for characterisation and reporting [27, 106]. While the review of common isolation methods is beyond the present scope and described in detail elsewhere [102, 107], the following section will discuss the application of immunoaffinity-based capture methods to detect cell- or tissue-specific EVs and analyse their cargo in the context of chronic liver diseases. Supplemented by emerging technologies, we envision this to serve as a foundation for the implementation of informed and actionable biomarker strategies with broader relevance to any condition or application. Studies described in this section are also listed in Table 2 under *Cargo detected in circulating EVs* for the particular disease.

## Technologies to assess tissue-specific EVs

Given the biogenesis pathway and cell of origin influence surface protein expression on EVs, the isolation of particular subpopulations can be achieved by immunoaffinity capture (IAC) [108]. IAC is based on the interactions of EV surface molecules with antibodies, most commonly against tetraspanins, that are covalently linked to a fixed phase, such as magnetic or non-magnetic beads, plastic or silica plates, porous monolithic microtips or microfluidic devices [102]. Compared to conventional isolation methods, the use of antibodies permits the extraction of highly pure and specific EVs, lending itself to customisation against EV markers of interest [107, 108]. For instance, ASGR and CYP2E1 are known membrane-localised EV constituents with high specificity for EVs of hepatocyte origin, and have been applied for the selective analysis of hepatocyte-derived biomarkers for liver disease in vivo [109]. However, targeting specific EV populations inherently reduces yield. Efficient recovery by IAC depends on the availability of antibodies with sufficient specificity and stability. The high cost of antibodies also limits scaling of capture protocols to large sample volumes [107]. Though some techniques can be performed directly from biofluids, IAC is usually preceded by global EV enrichment. Variability between different global isolation methods with respect to subtype enrichment and cargo [110], underscores the importance of confirming compatibility of a chosen method with downstream analyses. Despite present limitations, emerging immunoaffinity-based technologies show promise to improve clinical biomarker analyses in a robust, timely and cost-effective manner.

## Immunobead- or plate-based capture

The most common approach to selectively isolate EV subtypes involves incubation of EVs with antibodies conjugated to magnetic beads or on plates [111]. EVs positive for cell- or disease-specific surface markers can then be selectively removed from the mixture by magnetic forces or immobilisation on the plate surface. This may also be used to improve the purity of samples, pre-enriched by precipitation or ultracentrifugation, by targeting tetraspanins [107]. The method is compatible with downstream analyses, including polymerase chain reaction (PCR), for direct quantification of molecular cargo [111], however, tight covalent bonds make the elution of bead- or plate-bound EVs challenging. Use of low pH buffers can release intact EVs but may interfere with subsequent investigations of functional activity [108]. Nonetheless, for diagnostic purposes, pull-down of specific EV samples presents the opportunity to comprehensively interrogate cargo across multi-omics platforms and identify disease-specific molecular signatures. Immunoprecipitation of cell-specific EVs from biofluids has been applied in biomarker discovery by a select number of groups, in the context of neuronal pathology [112–115], cerebrovascular disease [116], transplant rejection [117], melanoma [118] and prostate cancer [119]. These reports consistently support the notion that EVs preferentially enriched for tissue origin are most informative of disease and thus enhance sensitivity and specificity of biomarker analyses. We recently isolated hepatocyte-derived EVs by anti-ASGR1 immunoprecipitation for the study of DMET induction by rifampicin and in pregnancy [53] and to compare the performance of miRNA biomarkers for NAFLD in unfractionated plasma, global circulating EVs and liver-specific EVs [68]. Only in applying the selective isolation technique, was a strong significant trend observed in biomarker expression with disease severity in NAFLD patients; thereby providing the first evidence for the utility of tissue-specific EV isolation techniques to improve diagnostic performance in chronic liver disease.

## Flow cytometry

Flow cytometry is a powerful technique that can be applied to the enumeration and sizing of EVs from biofluids and phenotyping of specific subpopulations [120]. Particles in suspension are passed through a laser beam and measured based on light scatter and fluorescent emission. Conventional flow cytometers were designed for measuring single cells, thus the limit of detection is substantially larger than the typical EV size distribution (between 200 and 500 nm depending on the instrument) [121]. Although modern developments in high-resolution flow cytometry have seen increased sensitivity towards lower limits (~ 100 nm), this still misses a significant portion of smaller EVs, as revealed by complementary techniques, such as nanoparticle tracking analysis (NTA) [122]. Alternatively, larger complexes can be formed using immunobeads to detect the smaller range of EVs. This is useful for detecting EV subtypes based on surface composition but provides no direct insight into vesicle size [121]. Multiplexed flow cytometry approaches allow the high-throughput analysis of multiple markers of interest [110, 123]; however, like other immunolabelling approaches (e.g. Fluorescent NTA and immunogold-label EM), cargo detection is restricted to surface expression.

In chronic liver disease, such as NAFLD, flow cytometry approaches uphold the potential for cell-specific circulating EVs to diagnose and track progression. In a diet-induced mouse model of NASH, Li et al. [33] followed changes in circulating EVs derived from hepatocytes (ASGR1 + , CYP2E1 +), macrophages (Galectin 3 +), neutrophils (Ly-6G/6C +) and platelets (CD61 +). Hepatocyte-specific EV levels were significantly elevated over the course of feeding, occurring prior to histological evidence of inflammation and correlated with NAFLD activity score and features of NASH, including lobular inflammation and ballooning. Similarly, macrophage- and neutrophil-derived EVs were increased and strongly associated with hepatic inflammation and fibrosis.

Povero et al. [3] also investigated changes in circulating hepatocyte-derived EVs, bearing ASGR1 and bile acyl-coenzyme A synthetase (SLC27A5), in human NASH patients with and without cirrhosis. SLC27A5 is a key enzyme in fatty acid uptake and synthesis. While greater expression is observed in steatotic hepatocytes, down-regulation has been associated with progression to cirrhosis due to loss of fat and functional parenchyma. In serum, SLC27A5 + EVs increased up to fourfold in NASH compared to healthy controls then decreased slightly in cirrhotic NASH. Meanwhile, ASGR1 + EV levels increased with disease severity, at almost twofold in pre-cirrhotic NASH and threefold in cirrhotic patients, compared to healthy controls. Liver-specific EV numbers exhibited strong correlations with features of NASH, including fibrosis stage, as well as various clinically relevant scores, such as FibroTest, Enhanced Liver Fibrosis (ELF) test and NAFLD Fibrosis Score (NFS). In addition, hepatocyte-derived EVs could predict clinically significant portal hypertension (hepatic venous pressure gradient [HVPG] ≥ 10 mmHg) in cirrhotic NASH patients with sensitivity of 92% and specificity of 75% (area under the receiver operating characteristic curve [AUROC] = 0.79), identifying the cut-off as ≥ 668 EVs/µl serum. Proteomic profiling also revealed several differentially expressed proteins that could distinguish advanced NASH from healthy controls (AUROC = 0.77) and pre-cirrhotic from cirrhotic NASH (AUROC = 0.80). Considering this analysis was performed on global circulating EVs and in late-stage NAFLD cohorts, the potential benefit of selectively analysing hepatocyte-derived EV protein cargo may be explored in simple steatosis or early NASH development.

It is noted that the above studies by and employed nanoscale flow cytometry with detection thresholds set to count EVs in the range of 110–1000 nm [33] and 200–1000 nm [3], respectively. Although limited to the larger EV range, earlier reports utilising conventional flow cytometers have also shown compelling results in support of tissue-specific EV biomarkers for chronic liver disease. Specifically, the profiling of immune cell-derived EVs discriminated patients with NAFL, NASH and HCV infection and healthy controls, and paralleled the extent of hepatic inflammation. Chronic HCV patients had greater circulating CD4 + and CD8 + T cell-derived EVs, while NAFLD patients had more EVs from invariant natural killer T cells and CD14 + macrophages. AUROC values ranged from 0.652 to 0.999 for various cohort pairs [124]. Later work demonstrated that the combination of surface markers, Annexin V, EpCAM, ASGR1 and CD133, could be used to identify tumour-associated EVs in circulation and distinguish between liver cancers (HCC and cholangiocarcinoma) and tumour-free cirrhosis [125]. Similarly, EVs expressing hepatocyte paraffin 1 (HepPar1) were found in great abundance in the circulation of patients with HCC, compared to virtually undetectable levels in tumour-free cirrhosis and healthy controls, and were proposed as an early marker of recurrence [126].

## Microfluidic devices

Recent innovations in microfluidic hardware have driven the development of compact chip-like devices for the detection and isolation of EVs from biofluids. Microfluidic devices sort particles through a network of microchannels of varying diameter, ranging from tens to hundreds of microns [102, 121]. Vesicle isolation is achieved either by actively applying electric, magnetic or acoustic forces, or in a passive manner, depending on immunoaffinity interactions and size exclusion [127]. In IA-based devices, antibody-functionalised surfaces immobilise target EVs flowing through the chip, to separate highly specific, pure vesicle subtypes. For example, the ExoChip device contains anti-CD63 to selectively capture exosomal small EV and has been applied for biomarker discovery in pancreatic cancer patients [128]. Progress in the design of these devices continue to improve sensitivity, reduce non-specific interactions and enhance capture efficiency by increasing surface area and mixing [127].

Key advantages of IA microfluidic chips include rapid processing time and low sample volume, requiring as little as ten microliters of plasma but taking up to a few hundred microliters [86]. It is noted that reducing sample input may be detrimental to downstream analyses, whereby biomarker yield in smaller volumes is insufficient for diagnostic purposes [102]. Despite their current complexity and cost, the development of integrated on-chip analysis of EV cargo positions microfluidic devices as promising novel tools for point of care testing (POCT) [129]. Captured EVs may be lysed by chemical (e.g. Triton X-100) or physical (e.g. electrical) means, and intraluminal nucleic acid amplified and analysed on-chip by quantitative PCR. Protein cargo may be detected by ELISA or released for off-chip proteomics [121, 127].

Recently, Sun et al. [32] designed an EV purification system, called EV Click Chips, to isolate HCC-derived EVs directly from 500 µl of plasma, via surface expression of ASGR1, EpCAM and CD147. Expression of 10 HCC-specific mRNA transcripts, analysed by digital droplet PCR in the captured EVs, gave exceptional diagnostic performance across several cohorts. Specifically, HCC was distinguished from all non-cancer (AUROC = 0.87) and from other primary malignancies (AUROC = 0.95) and early HCC detection could be achieved amongst at-risk cirrhosis patients of viral hepatitis, ALD or NASH aetiology (AUROC = 0.93), outperforming serum AFP (AUROC = 0.69). While the application of microfluidic devices is mainly at the proof-of-concept stage [111], longitudinal follow-up and validation in larger cohorts may soon see this novel non-invasive tool implemented in clinical settings for early diagnosis and patient monitoring.

Nano-plasmonic enhanced scattering assay.

Nano-plasmonic enhanced scattering assay (nPES) is a novel IAC technology that can isolate and quantify target EVs using capture and detection antibodies [104]. The assay, initially developed for tumour-derived EV from pancreatic cancer patients [130], was shown to considerably reduce cost, sample volume and analysis time and improved sensitivity compared to ELISA [104]. Similar to microfluidic devices, nPES is attractive for POCT given the assay consumes as little as 1–5 µl and can be performed directly from biofluids [13].

One research group recently developed a novel nPES assay to quantify hepatocyte-specific EVs as biomarkers for AH diagnosis [13] and for NAFLD resolution in obese patients undergoing weight loss surgery [109]. EV capture was achieved through ASGR2 or CYP2E1 and confirmed by CD63 positivity; and the capacity for circulating hepatocyte-specific EV levels to differentiate patients from controls was demonstrated in each cohort. Interestingly, hepatocyte-EVs correlated with steatosis and inflammation in NAFLD. The findings by Nakao and co-workers are in line with an earlier study that showed a 68% reduction in circulating hepatocyte-EVs (ASGR1 + , HepPar1 +) after bariatric surgery; however, the use of flow cytometry limited vesicle detection to those within 200–900 nm diameter [131].

Finally, lipidomic analysis revealed differential sphingolipid cargo in global EVs that was integrated in multivariable logistic regression models with MELD score and log global EV count to predict 90-day mortality in AH (AUC = 0.91) [13]; and with body mass index and small EV (110,000×*g* UC pellet) count to identify NAFLD (AUC = 0.80) [109]. Given the shortfalls of global EV analysis, further development and validation of nPES and other technologies to selectively analyse molecular signatures in cell-specific EVs may advance clinical translation of predictive models such as these.

### Concluding remarks

EVs mediate a vast array of complex biological functions, related to the maintenance of liver homeostasis as well as the initiation and progression of liver diseases. A multiplicity of reports underpins the mechanistic link between EV-mediated cellular crosstalk and pathogenic processes that translate to differential expression in EV-based biomarkers across human patients and healthy subjects. The stability and accessibility of EV in peripheral blood are among attractive characteristics that compel their application as minimally invasive biomarkers. In the field of chronic liver disease, such tools for diagnosis and tracking of disease status and response to therapeutic intervention is in critical demand. The advancement of platforms designed to specifically isolate and analyse EVs, derived from cells or tissues relevant to the condition of interest, may greatly enhance the sensitivity and reproducibility of circulating EV biomarker analyses. It is our overarching view that clinical use will be supported by the development of these technologies and a holistic approach to evaluating disease-specific EV signatures of composite molecular species.

## Data Availability

Enquiries about data availability should be directed to the authors.

## References

[1] Chen L, Chen R, Kemper S, Cong M, You H, Brigstock DR (2018). Therapeutic effects of serum extracellular vesicles in liver fibrosis. J Extracell Vesic.

[2] Moon AM, Singal AG, Tapper EB (2020). Contemporary epidemiology of chronic liver disease and cirrhosis. Clin Gastroenterol Hepatol.

[3] Povero D, Yamashita H, Ren W, Subramanian MG, Myers RP, Eguchi A, Simonetto DA, Goodman ZD, Harrison SA, Sanyal AJ, Bosch J, Feldstein AE (2020). Characterization and proteome of circulating extracellular vesicles as potential biomarkers for NASH. Hepatol Commun.

[4] Hernández A, Arab JP, Reyes D, Lapitz A, Moshage H, Bañales JM, Arrese M (2020). Extracellular vesicles in NAFLD/ALD: from pathobiology to therapy. Cells.

[5] Newman LA, Sorich MJ, Rowland A (2020). Role of extracellular vesicles in the pathophysiology, diagnosis and tracking of non-alcoholic fatty liver disease. J Clin Med.

[6] Wong MCS, Huang JLW, George J, Huang J, Leung C, Eslam M, Chan HLY, Ng SC (2019). The changing epidemiology of liver diseases in the Asia-Pacific region. Nat Rev Gastroenterol Hepatol.

[7] Li X, Chen R, Kemper S, Brigstock DR (2020). dynamic changes in function and proteomic composition of extracellular vesicles from hepatic stellate cells during cellular activation. Cells.

[8] Simon TG, Roelstraete B, Khalili H, Hagström H, Ludvigsson JF (2020) Mortality in biopsy-confirmed nonalcoholic fatty liver disease: results from a nationwide cohort. Gut. 10.1136/gutjnl-2020-322786 10.1136/gutjnl-2020-322786 PMC8185553 33037056

[9] Ando Y, Jou JH (2021). Nonalcoholic fatty liver disease and recent guideline updates. Clin Liver Dis.

[10] Almeida PH, Matielo CEL, Curvelo LA, Rocco RA, Felga G, Della Guardia B, Boteon YL (2021). Update on the management and treatment of viral hepatitis. World J Gastroenterol.

[11] Mann J, Reeves HL, Feldstein AE (2018). Liquid biopsy for liver diseases. Gut.

[12] Sumida Y, Nakajima A, Itoh Y (2014). Limitations of liver biopsy and non-invasive diagnostic tests for the diagnosis of nonalcoholic fatty liver disease/nonalcoholic steatohepatitis. World J Gastroenterol.

[13] Sehrawat TS, Arab JP, Liu M, Amrollahi P, Wan M, Fan J, Nakao Y, Pose E, Navarro-Corcuera A, Dasgupta D, Liao C-Y, He L, Mauer AS, Avitabile E, Ventura-Cots M, Bataller RA, Sanyal AJ, Chalasani NP, Heimbach JK, Watt KD, Gores GJ, Gines P, Kamath PS, Simonetto DA, Hu TY, Shah VH, Malhi H (2021). circulating extracellular vesicles carrying sphingolipid cargo for the diagnosis and dynamic risk profiling of alcoholic hepatitis. Hepatology.

[14] Wong VW-S, Adams LA, de Lédinghen V, Wong GL-H, Sookoian S (2018). Noninvasive biomarkers in NAFLD and NASH—current progress and future promise. Nat Rev Gastroenterol Hepatol.

[15] Petta S, Vanni E, Bugianesi E, Di Marco V, Camma C, Cabibi D, Mezzabotta L, Craxi A (2015). The combination of liver stiffness measurement and NAFLD fibrosis score improves the noninvasive diagnostic accuracy for severe liver fibrosis in patients with nonalcoholic fatty liver disease. Liver Int.

[16] Younossi Z, Tacke F, Arrese M, Chander Sharma B, Mostafa I, Bugianesi E, Wai-Sun Wong V, Yilmaz Y, George J, Fan J, Vos MB (2019). Global perspectives on nonalcoholic fatty liver disease and nonalcoholic steatohepatitis. Hepatology.

[17] Eguchi A, Lazaro RG, Wang J, Kim J, Povero D, Willliams B, Ho SB, Stärkel P, Schnabl B, Ohno-Machado L, Tsukamoto H, Feldstein AE (2017). Extracellular vesicles released by hepatocytes from gastric infusion model of alcoholic liver disease contain a MicroRNA barcode that can be detected in blood. Hepatology.

[18] Koeck ES, Iordanskaia T, Sevilla S, Ferrante SC, Hubal MJ, Freishtat RJ, Nadler EP (2014). Adipocyte exosomes induce transforming growth factor beta pathway dysregulation in hepatocytes: a novel paradigm for obesity-related liver disease. J Surg Res.

[19] Gho YS, Lee C (2017). Emergent properties of extracellular vesicles: a holistic approach to decode the complexity of intercellular communication networks. Mol BioSyst.

[20] Sung S, Kim J, Jung Y (2018). Liver-derived exosomes and their implications in liver pathobiology. Int J Mol Sci.

[21] Chen L, Charrier A, Zhou Y, Chen R, Yu B, Agarwal K, Tsukamoto H, Lee LJ, Paulaitis ME, Brigstock DR (2014). Epigenetic regulation of connective tissue growth factor by MicroRNA-214 delivery in exosomes from mouse or human hepatic stellate cells. Hepatology.

[22] Malhi H (2019) Emerging role of extracellular vesicles in liver diseases. Am J Physiol Gastrointest Liver Physiol 317(5):G739–G749. 10.1152/ajpgi.00183.2019 10.1152/ajpgi.00183.2019 PMC6879890 31545919

[23] Mathieu M, Martin-Jaular L, Lavieu G, Théry C (2019). Specificities of secretion and uptake of exosomes and other extracellular vesicles for cell-to-cell communication. Nat Cell Biol.

[24] Greening DW, Simpson RJ (2018) Understanding extracellular vesicle diversity—current status. Expert Rev Proteom. 10.1080/14789450.2018.1537788 10.1080/14789450.2018.1537788 30326765

[25] Tetta C, Bruno S, Fonsato V, Deregibus MC, Camussi G (2011). The role of microvesicles in tissue repair. Organogenesis.

[26] Kowal J, Arras G, Colombo M, Jouve M, Morath JP, Primdal-Bengtson B, Dingli F, Loew D, Tkach M, Théry C (2016). Proteomic comparison defines novel markers to characterize heterogeneous populations of extracellular vesicle subtypes. Proc Natl Acad Sci.

[27] Théry C, Witwer KW, Aikawa E, Alcaraz MJ, Anderson JD, Andriantsitohaina R, Antoniou A, Arab T, Archer F, Atkin-Smith GK, Ayre DC, Bach J-M, Bachurski D, Baharvand H, Balaj L, Baldacchino S, Bauer NN, Baxter AA, Bebawy M, Beckham C, Bedina Zavec A, Benmoussa A, Berardi AC, Bergese P, Bielska E, Blenkiron C, Bobis-Wozowicz S, Boilard E, Boireau W, Bongiovanni A, Borràs FE, Bosch S, Boulanger CM, Breakefield X, Breglio AM, Brennan MÁ, Brigstock DR, Brisson A, Broekman MLD, Bromberg JF, Bryl-Górecka P, Buch S, Buck AH, Burger D, Busatto S, Buschmann D, Bussolati B, Buzás EI, Byrd JB, Camussi G, Carter DRF, Caruso S, Chamley LW, Chang Y-T, Chen C, Chen S, Cheng L, Chin AR, Clayton A, Clerici SP, Cocks A, Cocucci E, Coffey RJ, Cordeiro-da-Silva A, Couch Y, Coumans FAW, Coyle B, Crescitelli R, Criado MF, D’Souza-Schorey C, Das S, Datta Chaudhuri A, de Candia P, De Santana EF, De Wever O, del Portillo HA, Demaret T, Deville S, Devitt A, Dhondt B, Di Vizio D, Dieterich LC, Dolo V, Dominguez Rubio AP, Dominici M, Dourado MR, Driedonks TAP, Duarte FV, Duncan HM, Eichenberger RM, Ekström K, El Andaloussi S, Elie-Caille C, Erdbrügger U, Falcón-Pérez JM, Fatima F, Fish JE, Flores-Bellver M, Försönits A, Frelet-Barrand A, Fricke F, Fuhrmann G, Gabrielsson S, Gámez-Valero A, Gardiner C, Gärtner K, Gaudin R, Gho YS, Giebel B, Gilbert C, Gimona M, Giusti I, Goberdhan DCI, Görgens A, Gorski SM, Greening DW, Gross JC, Gualerzi A, Gupta GN, Gustafson D, Handberg A, Haraszti RA, Harrison P, Hegyesi H, Hendrix A, Hill AF, Hochberg FH, Hoffmann KF, Holder B, Holthofer H, Hosseinkhani B, Hu G, Huang Y, Huber V, Hunt S, Ibrahim AG-E, Ikezu T, Inal JM, Isin M, Ivanova A, Jackson HK, Jacobsen S, Jay SM, Jayachandran M, Jenster G, Jiang L, Johnson SM, Jones JC, Jong A, Jovanovic-Talisman T, Jung S, Kalluri R, Kano S-I, Kaur S, Kawamura Y, Keller ET, Khamari D, Khomyakova E, Khvorova A, Kierulf P, Kim KP, Kislinger T, Klingeborn M, Klinke DJ, Kornek M, Kosanović MM, Kovács ÁF, Krämer-Albers E-M, Krasemann S, Krause M, Kurochkin IV, Kusuma GD, Kuypers S, Laitinen S, Langevin SM, Languino LR, Lannigan J, Lässer C, Laurent LC, Lavieu G, Lázaro-Ibáñez E, Le Lay S, Lee M-S, Lee YXF, Lemos DS, Lenassi M, Leszczynska A, Li ITS, Liao K, Libregts SF, Ligeti E, Lim R, Lim SK, Linē A, Linnemannstöns K, Llorente A, Lombard CA, Lorenowicz MJ, Lörincz ÁM, Lötvall J, Lovett J, Lowry MC, Loyer X, Lu Q, Lukomska B, Lunavat TR, Maas SLN, Malhi H, Marcilla A, Mariani J, Mariscal J, Martens-Uzunova ES, Martin-Jaular L, Martinez MC, Martins VR, Mathieu M, Mathivanan S, Maugeri M, McGinnis LK, McVey MJ, Meckes DG, Meehan KL, Mertens I, Minciacchi VR, Möller A, Møller Jørgensen M, Morales-Kastresana A, Morhayim J, Mullier F, Muraca M, Musante L, Mussack V, Muth DC, Myburgh KH, Najrana T, Nawaz M, Nazarenko I, Nejsum P, Neri C, Neri T, Nieuwland R, Nimrichter L, Nolan JP, Nolte-’t Hoen ENM, Noren Hooten N, O’Driscoll L, O’Grady T, O’Loghlen A, Ochiya T, Olivier M, Ortiz A, Ortiz LA, Osteikoetxea X, Østergaard O, Ostrowski M, Park J, Pegtel DM, Peinado H, Perut F, Pfaffl MW, Phinney DG, Pieters BCH, Pink RC, Pisetsky DS, Pogge von Strandmann E, Polakovicova I, Poon IKH, Powell BH, Prada I, Pulliam L, Quesenberry P, Radeghieri A, Raffai RL, Raimondo S, Rak J, Ramirez MI, Raposo G, Rayyan MS, Regev-Rudzki N, Ricklefs FL, Robbins PD, Roberts DD, Rodrigues SC, Rohde E, Rome S, Rouschop KMA, Rughetti A, Russell AE, Saá P, Sahoo S, Salas-Huenuleo E, Sánchez C, Saugstad JA, Saul MJ, Schiffelers RM, Schneider R, Schøyen TH, Scott A, Shahaj E, Sharma S, Shatnyeva O, Shekari F, Shelke GV, Shetty AK, Shiba K, Siljander PRM, Silva AM, Skowronek A, Snyder OL, Soares RP, Sódar BW, Soekmadji C, Sotillo J, Stahl PD, Stoorvogel W, Stott SL, Strasser EF, Swift S, Tahara H, Tewari M, Timms K, Tiwari S, Tixeira R, Tkach M, Toh WS, Tomasini R, Torrecilhas AC, Tosar JP, Toxavidis V, Urbanelli L, Vader P, van Balkom BWM, van der Grein SG, Van Deun J, van Herwijnen MJC, Van Keuren-Jensen K, van Niel G, van Royen ME, van Wijnen AJ, Vasconcelos MH, Vechetti IJ, Veit TD, Vella LJ, Velot É, Verweij FJ, Vestad B, Viñas JL, Visnovitz T, Vukman KV, Wahlgren J, Watson DC, Wauben MHM, Weaver A, Webber JP, Weber V, Wehman AM, Weiss, DJ Welsh, JA Wendt, S, Wheelock AM, Wiener Z, Witte, L Wolfram, J, Xagorari A, Xander, P Xu, J Yan, X Yáñez-Mó, M Yin, H Yuana, Y, Zappulli V, Zarubova J, Žėkas V, Zhang J-y, Zhao Z, Zheng L, Zheutlin AR, Zickler AM, Zimmermann P, Zivkovic AM, Zocco D, Zuba-Surma EK (2018) Minimal information for studies of extracellular vesicles 2018 (MISEV2018): a position statement of the International Society for Extracellular Vesicles and update of the MISEV2014 guidelines. J Extracell Vesicles **7**(1):5750. 10.1080/20013078.2018.1535750 10.1080/20013078.2018.1535750 PMC6322352 30637094

[28] Jeppesen DK, Fenix AM, Franklin JL, Higginbotham JN, Zhang Q, Zimmerman LJ, Liebler DC, Ping J, Liu Q, Evans R, Fissell WH, Patton JG, Rome LH, Burnette DT, Coffey RJ (2019). Reassessment of exosome composition. Cell.

[29] Larssen P, Wik L, Czarnewski P, Eldh M, Löf L, Ronquist KG, Dubois L, Freyhult E, Gallant CJ, Oelrich J, Larsson A, Ronquist G, Villablanca EJ, Landegren U, Gabrielsson S, Kamali-Moghaddam M (2017). tracing cellular origin of human exosomes using multiplex proximity extension assays*. Mol Cell Proteomics.

[30] Kostallari E, Hirsova P, Prasnicka A, Verma VK, Yaqoob U, Wongjarupong N, Roberts LR, Shah VH (2018). Hepatic stellate cell–derived platelet-derived growth factor receptor-alpha-enriched extracellular vesicles promote liver fibrosis in mice through SHP2. Hepatology.

[31] Dasgupta D, Nakao Y, Mauer AS, Thompson JM, Sehrawat TS, Liao C-Y, Krishnan A, Lucien F, Guo Q, Liu M, Xue F, Fukushima M, Katsumi T, Bansal A, Pandey MK, Maiers JL, DeGrado T, Ibrahim SH, Revzin A, Pavelko KD, Barry MA, Kaufman RJ, Malhi H (2020). IRE1A stimulates hepatocyte-derived extracellular vesicles that promote inflammation in mice with steatohepatitis. Gastroenterology.

[32] Sun N, Lee Y-T, Zhang RY, Kao R, Teng P-C, Yang Y, Yang P, Wang JJ, Smalley M, Chen P-J, Kim M, Chou S-J, Bao L, Wang J, Zhang X, Qi D, Palomique J, Nissen N, Han S-HB, Sadeghi S, Finn RS, Saab S, Busuttil RW, Markovic D, Elashoff D, Yu H-H, Li H, Heaney AP, Posadas E, You S, Yang JD, Pei R, Agopian VG, Tseng H-R, Zhu Y (2020). Purification of HCC-specific extracellular vesicles on nanosubstrates for early HCC detection by digital scoring. Nat Commun.

[33] Li J, Liu H, Mauer AS, Lucien F, Raiter A, Bandla H, Mounajjed T, Yin Z, Glaser KJ, Yin M, Malhi H (2019). Characterization of cellular sources and circulating levels of extracellular vesicles in a dietary murine model of nonalcoholic steatohepatitis. Hepatol Commun.

[34] Shah R, Patel T, Freedman JE (2018). circulating extracellular vesicles in human disease. N Engl J Med.

[35] Bruno S, Chiabotto G, Camussi G (2020). extracellular vesicles: a therapeutic option for liver fibrosis. Int J Mol Sci.

[36] Szabo G (2017). Momen-Heravi F (2017) Extracellular vesicles in liver disease and potential as biomarkers and therapeutic targets. Nat Rev Gastroenterol Hepatol.

[37] Azparren-Angulo M, Royo F, Gonzalez E, Liebana M, Brotons B, Berganza J, Goñi-de-Cerio F, Manicardi N, Abad-Jordà L, Gracia-Sancho J, Falcon-Perez JM (2021). Extracellular vesicles in hepatology: physiological role, involvement in pathogenesis, and therapeutic opportunities. Pharmacol Ther.

[38] Giugliano S, Kriss M, Golden-Mason L, Dobrinskikh E, Stone AEL, Soto-Gutierrez A, Mitchell A, Khetani SR, Yamane D, Stoddard M, Li H, Shaw GM, Edwards MG, Lemon SM, Gale M, Shah VH, Rosen HR (2015). Hepatitis C virus infection induces autocrine interferon signaling by human liver endothelial cells and release of exosomes, which inhibits viral replication. Gastroenterology.

[39] Conde-Vancells J, Rodriguez-Suarez E, Embade N, Gil D, Matthiesen R, Valle M, Elortza F, Lu SC, Mato JM, Falcon-Perez JM (2008). Characterization and comprehensive proteome profiling of exosomes secreted by hepatocytes. J Proteome Res.

[40] Royo F, Moreno L, Mleczko J, Palomo L, Gonzalez E, Cabrera D, Cogolludo A, Vizcaino FP, Van-Liempd S, Falcon-Perez JM (2017). Hepatocyte-secreted extracellular vesicles modify blood metabolome and endothelial function by an arginase-dependent mechanism. Sci Rep.

[41] Cai S, Cheng X, Pan X, Li J (2017). Emerging role of exosomes in liver physiology and pathology. Hepatol Res.

[42] Nojima H, Freeman CM, Schuster RM, Japtok L, Kleuser B, Edwards MJ, Gulbins E, Lentsch AB (2016). Hepatocyte exosomes mediate liver repair and regeneration via sphingosine-1-phosphate. J Hepatol.

[43] Wang R, Ding Q, Yaqoob U, de Assuncao TM, Verma VK, Hirsova P, Cao S, Mukhopadhyay D, Huebert RC, Shah VH (2015). Exosome adherence and internalization by hepatic stellate cells triggers sphingosine 1-phosphate-dependent migration. J Biol Chem.

[44] Chen L, Chen R, Velazquez VM, Brigstock DR (2016). Fibrogenic signaling is suppressed in hepatic stellate cells through targeting of connective tissue growth factor (CCN2) by cellular or exosomal MicroRNA-199a-5p. Am J Pathol.

[45] Chen, L., Chen, R., Kemper, S., Charrier, A., and Brigstock, D.R., *Suppression of fibrogenic signaling in hepatic stellate cells by Twist1-dependent microRNA-214 expression: Role of exosomes in horizontal transfer of Twist1.* American journal of physiology. Gastrointestinal and liver physiology, 2015. **309**(6): G491-G499. 10.1152/ajpgi.00140.2015 10.1152/ajpgi.00140.2015 PMC4572411 26229009

[46] Charrier A, Chen R, Chen L, Kemper S, Hattori T, Takigawa M, Brigstock DR (2014). Exosomes mediate intercellular transfer of pro-fibrogenic connective tissue growth factor (CCN2) between hepatic stellate cells, the principal fibrotic cells in the liver. Surgery.

[47] Li X, Liu R, Huang Z, Gurley EC, Wang X, Wang J, He H, Yang H, Lai G, Zhang L, Bajaj JS, White M, Pandak WM, Hylemon PB, Zhou H (2018). Cholangiocyte-derived exosomal long noncoding RNA H19 promotes cholestatic liver injury in mouse and humans. Hepatology.

[48] Witek RP, Yang L, Liu R, Jung Y, Omenetti A, Syn WK, Choi SS, Cheong Y, Fearing CM, Agboola KM, Chen W, Diehl AM (2009). Liver cell-derived microparticles activate hedgehog signaling and alter gene expression in hepatic endothelial cells. Gastroenterology.

[49] Achour B, Al-Majdoub ZM, Grybos-Gajniak A, Lea K, Kilford P, Zhang M, Knight D, Barber J, Schageman J, Rostami-Hodjegan A (2021). Liquid biopsy enables quantification of the abundance and interindividual variability of hepatic enzymes and transporters. Clin Pharmacol Ther.

[50] Rodrigues A, Rowland A (2019). From endogenous compounds as biomarkers to plasma-derived nanovesicles as liquid biopsy; has the golden age of translational PK-ADME-DDI science finally arrived?. Clin Pharmacol Ther.

[51] Kumar S, Sinha N, Gerth KA, Rahman MA, Yallapu MM, Midde NM (2017). Specific packaging and circulation of cytochromes P450, especially 2E1 isozyme, in human plasma exosomes and their implications in cellular communications. Biochem Biophys Res Commun.

[52] Rowland A, Ruanglertboon W, van Dyk M, Wijayakumara D, Wood LS, Meech R, Mackenzie PI, Rodrigues AD, Marshall J-C, Sorich MJ (2019). Plasma extracellular nanovesicle (exosome)-derived biomarkers for drug metabolism pathways: a novel approach to characterize variability in drug exposure. Br J Clin Pharmacol.

[53] Rodrigues AD, van Dyk M, Sorich MJ, Fahmy A, Useckaite Z, Newman LA, Kapetas AJ, Mounzer R, Wood LS, Johnson JG, Rowland A (2021). Exploring the use of serum-derived small extracellular vesicles as liquid biopsy to study the induction of hepatic cytochromes P450 and organic anion transporting polypeptides. Clin Pharmacol Ther.

[54] Gerth K, Kodidela S, Mahon M, Haque S, Verma N, Kumar S (2019). Circulating extracellular vesicles containing xenobiotic metabolizing CYP enzymes and their potential roles in extrahepatic cells via cell–cell interactions. Int J Mol Sci.

[55] Jamwal R, Barlock BJ (2020) Nonalcoholic fatty liver disease (NAFLD) and hepatic cytochrome P450 (CYP) enzymes. Pharmaceuticals 13(9). 10.3390/ph13090222 10.3390/ph13090222 PMC7560175 32872474

[56] Srinivas AN, Suresh D, Santhekadur PK, Suvarna D, Kumar DP (2020). Extracellular vesicles as inflammatory drivers in NAFLD. Front Immunol.

[57] Eslam M, Sanyal AJ, George J, Sanyal A, Neuschwander-Tetri B, Tiribelli C, Kleiner DE, Brunt E, Bugianesi E, Yki-Järvinen H, Grønbæk H, Cortez-Pinto H, George J, Fan J, Valenti L, Abdelmalek M, Romero-Gomez M, Rinella M, Arrese M, Eslam M, Bedossa P, Newsome PN, Anstee QM, Jalan R, Bataller R, Loomba R, Sookoian S, Sarin SK, Harrison S, Kawaguchi T, Wong VW-S, Ratziu V, Yilmaz Y, Younossi Z (2020). MAFLD: a consensus-driven proposed nomenclature for metabolic associated fatty liver disease. Gastroenterology.

[58] Eslam M, Newsome PN, Sarin SK, Anstee QM, Targher G, Romero-Gomez M, Zelber-Sagi S, Wai-Sun Wong V, Dufour J-F, Schattenberg JM, Kawaguchi T, Arrese M, Valenti L, Shiha G, Tiribelli C, Yki-Järvinen H, Fan J-G, Grønbæk H, Yilmaz Y, Cortez-Pinto H, Oliveira CP, Bedossa P, Adams LA, Zheng M-H, Fouad Y, Chan W-K, Mendez-Sanchez N, Ahn SH, Castera L, Bugianesi E, Ratziu V, George J (2020). A new definition for metabolic dysfunction-associated fatty liver disease: an international expert consensus statement. J Hepatol.

[59] Suzuki A, Diehl AM (2017). Nonalcoholic steatohepatitis. Annu Rev Med.

[60] Jonas W, Schürmann A (2020) Genetic and epigenetic factors determining NAFLD risk. Mol Metab 101111. 10.1016/j.molmet.2020.101111 10.1016/j.molmet.2020.101111 PMC8324682 33160101

[61] Zhang X, Ji X, Wang Q, Li JZ (2018). New insight into inter-organ crosstalk contributing to the pathogenesis of non-alcoholic fatty liver disease (NAFLD). Protein Cell.

[62] Hirsova P, Ibrahim SH, Krishnan A, Verma VK, Bronk SF, Werneburg NW, Charlton MR, Shah VH, Malhi H, Gores GJ (2016). Lipid-induced signaling causes release of inflammatory extracellular vesicles from hepatocytes. Gastroenterology.

[63] Ibrahim SH, Gores GJ, Hirsova P, Kirby M, Miles L, Jaeschke A, Kohli R (2014). Mixed lineage kinase 3 deficient mice are protected against the high fat high carbohydrate diet-induced steatohepatitis. Liver Int.

[64] Ibrahim SH, Hirsova P, Tomita K, Bronk SF, Werneburg NW, Harrison SA, Goodfellow VS, Malhi H, Gores GJ (2016). Mixed lineage kinase 3 mediates release of C-X-C motif ligand 10-bearing chemotactic extracellular vesicles from lipotoxic hepatocytes. Hepatology.

[65] Kakazu E, Mauer AS, Yin M, Malhi H (2016). Hepatocytes release ceramide-enriched pro-inflammatory extracellular vesicles in an IRE1α-dependent manner. J Lipid Res.

[66] Povero D, Panera N, Eguchi A, Johnson CD, Papouchado BG, de Araujo Horcel L, Pinatel EM, Alisi A, Nobili V, Feldstein AE (2015). Lipid-induced hepatocyte-derived extracellular vesicles regulate hepatic stellate cell via microRNAs targeting PPAR-γ. Cell Mol Gastroenterol Hepatol.

[67] Povero D, Eguchi A, Niesman IR, Andronikou N, de Mollerat du Jeu, X., Mulya, A., Berk, M., Lazic, M., Thapaliya, S., Parola, M., Patel, H.H., Feldstein, A.E. (2013). Lipid-induced toxicity stimulates hepatocytes to release angiogenic microparticles that require Vanin-1 for uptake by endothelial cells. Sci Signal.

[68] Newman LA, Useckaite Z, Johnson J, Sorich MJ, Hopkins AM, Rowland A (2022). Selective isolation of liver-derived extracellular vesicles redefines performance of miRNA biomarkers for non-alcoholic fatty liver disease. Biomedicines.

[69] Prasun P, Ginevic I, Oishi K (2021). Mitochondrial dysfunction in nonalcoholic fatty liver disease and alcohol related liver disease. Transl Gastroenterol Hepatol.

[70] Garcia-Martinez I, Santoro N, Chen Y, Hoque R, Ouyang X, Caprio S, Shlomchik MJ, Coffman RL, Candia A, Mehal WZ (2016). Hepatocyte mitochondrial DNA drives nonalcoholic steatohepatitis by activation of TLR9. J Clin Investig.

[71] Kranendonk ME, Visseren FL, van Herwaarden JA, Nolte-'t Hoen EN, de Jager W, Wauben MH, Kalkhoven E (2014). Effect of extracellular vesicles of human adipose tissue on insulin signaling in liver and muscle cells. Obesity (Silver Spring).

[72] Thomou T, Mori MA, Dreyfuss JM, Konishi M, Sakaguchi M, Wolfrum C, Rao TN, Winnay JN, Garcia-Martin R, Grinspoon SK, Gorden P, Kahn CR (2017). Adipose-derived circulating miRNAs regulate gene expression in other tissues. Nature.

[73] Keinicke H, Sun G, Mentzel CMJ, Fredholm M, John LM, Andersen B, Raun K, Kjaergaard M (2020). FGF21 regulates hepatic metabolic pathways to improve steatosis and inflammation. Endocr Connect.

[74] Sharma P, Arora A (2020). Clinical presentation of alcoholic liver disease and non-alcoholic fatty liver disease: spectrum and diagnosis. Transl Gastroenterol Hepatol.

[75] Eguchi A, Yan R, Pan SQ, Wu R, Kim J, Chen Y, Ansong C, Smith RD, Tempaku M, Ohno-Machado L, Takei Y, Feldstein AE, Tsukamoto H (2020). Comprehensive characterization of hepatocyte-derived extracellular vesicles identifies direct miRNA-based regulation of hepatic stellate cells and DAMP-based hepatic macrophage IL-1β and IL-17 upregulation in alcoholic hepatitis mice. J Mol Med.

[76] Ma J, Cao H, Rodrigues RM, Xu M, Ren T, He Y, Hwang S, Feng D, Ren R, Yang P, Liangpunsakul S, Sun J, Gao B (2020). Chronic-plus-binge alcohol intake induces production of proinflammatory mtDNA-enriched extracellular vesicles and steatohepatitis via ASK1/p38MAPKα-dependent mechanisms. JCI Insight.

[77] Verma VK, Li H, Wang R, Hirsova P, Mushref M, Liu Y, Cao S, Contreras PC, Malhi H, Kamath PS, Gores GJ, Shah VH (2016). Alcohol stimulates macrophage activation through caspase-dependent hepatocyte derived release of CD40L containing extracellular vesicles. J Hepatol.

[78] Momen-Heravi F, Bala S, Kodys K, Szabo G (2015). Exosomes derived from alcohol-treated hepatocytes horizontally transfer liver specific miRNA-122 and sensitize monocytes to LPS. Sci Rep.

[79] Bala S, Petrasek J, Mundkur S, Catalano D, Levin I, Ward J, Alao H, Kodys K, Szabo G (2012). Circulating microRNAs in exosomes indicate hepatocyte injury and inflammation in alcoholic, drug-induced, and inflammatory liver diseases. Hepatology.

[80] Zamor PJ, deLemos AS, Russo MW (2017) Viral hepatitis and hepatocellular carcinoma: etiology and management. J Gastrointest Oncol 8(2):229–242. 10.21037/jgo.2017.03.14 10.21037/jgo.2017.03.14 PMC5401856 28480063

[81] Kakizaki M, Yamamoto Y, Yabuta S, Kurosaki N, Kagawa T, Kotani A (2019). The immunological function of extracellular vesicles in hepatitis B virus-infected hepatocytes. PLoS ONE.

[82] Deng L, Jiang W, Wang X, Merz A, Hiet M-S, Chen Y, Pan X, Jiu Y, Yang Y, Yu B, He Y, Tu Z, Niu J, Bartenschlager R, Long G (2019). Syntenin regulates hepatitis C virus sensitivity to neutralizing antibody by promoting E2 secretion through exosomes. J Hepatol.

[83] Bukong TN, Momen-Heravi F, Kodys K, Bala S, Szabo G (2014). Exosomes from hepatitis C infected patients transmit HCV infection and contain replication competent viral RNA in complex with Ago2-miR122-HSP90. PLoS Pathog.

[84] Devhare PB, Sasaki R, Shrivastava S, Di Bisceglie AM, Ray R, Ray RB (2017). Exosome-mediated intercellular communication between Hepatitis C virus-infected hepatocytes and hepatic stellate cells. J Virol.

[85] Kornek M, Popov Y, Libermann TA, Afdhal NH, Schuppan D (2011) Human T cell microparticles circulate in blood of hepatitis patients and induce fibrolytic activation of hepatic stellate cells. Hepatology (Baltimore, Md.) 53(1):230–242. 10.1002/hep.23999 10.1002/hep.23999 PMC3505073 20979056

[86] Lee YT, Tran BV, Wang JJ, Liang IY, You S, Zhu Y, Agopian VG, Tseng HR, Yang JD (2021) The role of extracellular vesicles in disease progression and detection of hepatocellular carcinoma*.* Cancers 13(12). 10.3390/cancers13123076 10.3390/cancers13123076 PMC8233859 34203086

[87] Lubel JS, Roberts SK, Strasser SI, Thompson AJ, Philip J, Goodwin M, Clarke S, Crawford DHG, Levy MT, Shackel N (2021). Australian recommendations for the management of hepatocellular carcinoma: a consensus statement. Med J Aust.

[88] Marrero JA, Kulik LM, Sirlin CB, Zhu AX, Finn RS, Abecassis MM, Roberts LR, Heimbach JK (2018). Diagnosis, staging, and management of hepatocellular carcinoma: 2018 practice guidance by the American association for the study of liver diseases. Hepatology.

[89] Xue X, Wang X, Zhao Y, Hu R, Qin L (2018). Exosomal miR-93 promotes proliferation and invasion in hepatocellular carcinoma by directly inhibiting TIMP2/TP53INP1/CDKN1A. Biochem Biophys Res Commun.

[90] Cui Y, Xu H-F, Liu M-Y, Xu Y-J, He J-C, Zhou Y, Cang S-D (2019). Mechanism of exosomal microRNA-224 in development of hepatocellular carcinoma and its diagnostic and prognostic value. World J Gastroenterol.

[91] Qu Z, Wu J, Wu J, Ji A, Qiang G, Jiang Y, Jiang C, Ding Y (2017) Exosomal miR-665 as a novel minimally invasive biomarker for hepatocellular carcinoma diagnosis and prognosis. Oncotarget 8(46):80666–80678. 10.18632/oncotarget.20881 10.18632/oncotarget.20881 PMC5655229 29113334

[92] Tang J, Li Y, Liu K, Zhu Q, Yang WH, Xiong LK, Guo DL (2018) Exosomal miR-9–3p suppresses HBGF-5 expression and is a functional biomarker in hepatocellular carcinoma. Minerva Med 109(1):15–23. 10.23736/s0026-4806.17.05167-9 10.23736/S0026-4806.17.05167-9 28750499

[93] Shi M, Jiang Y, Yang L, Yan S, Wang YG, Lu XJ (2018). Decreased levels of serum exosomal miR-638 predict poor prognosis in hepatocellular carcinoma. J Cell Biochem.

[94] Sugimachi K, Matsumura T, Hirata H, Uchi R, Ueda M, Ueo H, Shinden Y, Iguchi T, Eguchi H, Shirabe K, Ochiya T, Maehara Y, Mimori K (2015). Identification of a bona fide microRNA biomarker in serum exosomes that predicts hepatocellular carcinoma recurrence after liver transplantation. Br J Cancer.

[95] Wang G, Zhao W, Wang H, Qiu G, Jiang Z, Wei G, Li X (2019) Exosomal MiR-744 inhibits proliferation and sorafenib chemoresistance in hepatocellular carcinoma by targeting PAX2. Med Sci Monit 25:7209–7217. 10.12659/MSM.919219 10.12659/MSM.919219 PMC6777417 31553714

[96] Kogure T, Lin W-L, Yan IK, Braconi C, Patel T (2011). Intercellular nanovesicle-mediated microRNA transfer: a mechanism of environmental modulation of hepatocellular cancer cell growth. Hepatology.

[97] He X, Yu J, Xiong L, Liu Y, Fan L, Li Y, Chen B, Chen J, Xu X (2019). Exosomes derived from liver cancer cells reprogram biological behaviors of LO2 cells by transferring Linc-ROR. Gene.

[98] He M, Qin H, Poon TCW, Sze S-C, Ding X, Co NN, Ngai S-M, Chan T-F, Wong N (2015). Hepatocellular carcinoma-derived exosomes promote motility of immortalized hepatocyte through transfer of oncogenic proteins and RNAs. Carcinogenesis.

[99] Fang T, Lv H, Lv G, Li T, Wang C, Han Q, Yu L, Su B, Guo L, Huang S, Cao D, Tang L, Tang S, Wu M, Yang W, Wang H (2018). Tumor-derived exosomal miR-1247-3p induces cancer-associated fibroblast activation to foster lung metastasis of liver cancer. Nat Commun.

[100] Arbelaiz A, Azkargorta M, Krawczyk M, Santos-Laso A, Lapitz A, Perugorria MJ, Erice O, Gonzalez E, Jimenez-Agüero R, Lacasta A, Ibarra C, Sanchez-Campos A, Jimeno JP, Lammert F, Milkiewicz P, Marzioni M, Macias RIR, Marin JJG, Patel T, Gores GJ, Martinez I, Elortza F, Falcon-Perez JM, Bujanda L, Banales JM (2017). Serum extracellular vesicles contain protein biomarkers for primary sclerosing cholangitis and cholangiocarcinoma. Hepatology.

[101] Unalp-Arida A, Ruhl CE (2017). Liver fibrosis scores predict liver disease mortality in the United States population. Hepatology.

[102] Konoshenko MY, Lekchnov EA, Vlassov AV, Laktionov PP (2018). Isolation of extracellular vesicles: general methodologies and latest trends. Biomed Res Int.

[103] Balaphas A, Meyer J, Sadoul R, Morel P, Gonelle-Gispert C, Bühler LH (2019). Extracellular vesicles: future diagnostic and therapeutic tools for liver disease and regeneration. Liver Int.

[104] Rojalin T, Phong B, Koster HJ, Carney RP (2019). Nanoplasmonic approaches for sensitive detection and molecular characterization of extracellular vesicles. Front Chem.

[105] Useckaite Z, Rodrigues AD, Hopkins AM, Newman LA, Johnson JG, Sorich MJ, Rowland A (2021) Role of extracellular vesicle derived biomarkers in drug metabolism and disposition. Drug Metab Dispos. 10.1124/dmd.121.000411 10.1124/dmd.121.000411 34353847

[106] Newman LA, Fahmy A, Sorich MJ, Best OG, Rowland A, Useckaite Z (2021). Importance of between and within subject variability in extracellular vesicle abundance and cargo when performing biomarker analyses. Cells.

[107] Sidhom K, Obi PO, Saleem A (2020). A review of exosomal isolation methods: is size exclusion chromatography the best option?. Int J Mol Sci.

[108] Mitchell MI, Ben-Dov IZ, Liu C, Ye K, Chow K, Kramer Y, Gangadharan A, Park S, Fitzgerald S, Ramnauth A, Perlin DS, Donato M, Bhoy E, Manouchehri Doulabi E, Poulos M, Kamali-Moghaddam M, Loudig O (2021). Extracellular Vesicle Capture by AnTibody of CHoice and Enzymatic Release (EV-CATCHER): a customizable purification assay designed for small-RNA biomarker identification and evaluation of circulating small-EVs. J Extracell Vesicles.

[109] Nakao Y, Amrollahi P, Parthasarathy G, Mauer AS, Sehrawat TS, Vanderboom P, Nair KS, Nakao K, Allen AM, Hu TY, Malhi H (2021). Circulating extracellular vesicles are a biomarker for NAFLD resolution and response to weight loss surgery. Nanomed Nanotechnol Biol Med.

[110] Mastoridis S, Bertolino GM, Whitehouse G, Dazzi F, Sanchez-Fueyo A, Martinez-Llordella M (2018). Multiparametric analysis of circulating exosomes and other small extracellular vesicles by advanced imaging flow cytometry. Front Immunol.

[111] Zarovni N, Corrado A, Guazzi P, Zocco D, Lari E, Radano G, Muhhina J, Fondelli C, Gavrilova J, Chiesi A (2015). Integrated isolation and quantitative analysis of exosome shuttled proteins and nucleic acids using immunocapture approaches. Methods.

[112] Mustapic M, Eitan E, Werner JK, Berkowitz ST, Lazaropoulos MP, Tran J, Goetzl EJ, Kapogiannis D (2017). Plasma extracellular vesicles enriched for neuronal origin: a potential window into brain pathologic processes. Front Neurosci.

[113] Shi M, Liu C, Cook TJ, Bullock KM, Zhao Y, Ginghina C, Li Y, Aro P, Dator R, He C, Hipp MJ, Zabetian CP, Peskind ER, Hu S-C, Quinn JF, Galasko DR, Banks WA, Zhang J (2014). Plasma exosomal α-synuclein is likely CNS-derived and increased in Parkinson's disease. Acta Neuropathol.

[114] Fiandaca MS, Kapogiannis D, Mapstone M, Boxer A, Eitan E, Schwartz JB, Abner EL, Petersen RC, Federoff HJ, Miller BL, Goetzl EJ (2015). Identification of preclinical Alzheimer's disease by a profile of pathogenic proteins in neurally derived blood exosomes: a case-control study. Alzheimers Dement.

[115] Goetzl EJ, Kapogiannis D, Schwartz JB, Lobach IV, Goetzl L, Abner EL, Jicha GA, Karydas AM, Boxer A, Miller BL (2016). Decreased synaptic proteins in neuronal exosomes of frontotemporal dementia and Alzheimer's disease. FASEB J.

[116] Goetzl EJ, Schwartz JB, Mustapic M, Lobach IV, Daneman R, Abner EL, Jicha GA (2017). Altered cargo proteins of human plasma endothelial cell-derived exosomes in atherosclerotic cerebrovascular disease. Faseb j.

[117] Vallabhajosyula P, Korutla L, Habertheuer A, Yu M, Rostami S, Yuan CX, Reddy S, Liu C, Korutla V, Koeberlein B, Trofe-Clark J, Rickels MR, Naji A (2017). Tissue-specific exosome biomarkers for noninvasively monitoring immunologic rejection of transplanted tissue. J Clin Invest.

[118] Sharma P, Ludwig S, Muller L, Hong CS, Kirkwood JM, Ferrone S, Whiteside TL (2018). Immunoaffinity-based isolation of melanoma cell-derived exosomes from plasma of patients with melanoma. J Extracell Vesicles.

[119] Mizutani K, Terazawa R, Kameyama K, Kato T, Horie K, Tsuchiya T, Seike K, Ehara H, Fujita Y, Kawakami K, Ito M, Deguchi T (2014). Isolation of prostate cancer-related exosomes. Anticancer Res.

[120] Welsh JA, Van Der Pol E, Arkesteijn GJA, Bremer M, Brisson A, Coumans F, Dignat-George F, Duggan E, Ghiran I, Giebel B, Görgens A, Hendrix A, Lacroix R, Lannigan J, Libregts SFWM, Lozano-Andrés E, Morales-Kastresana A, Robert S, De Rond L, Tertel T, Tigges J, De Wever O, Yan X, Nieuwland R, Wauben MHM, Nolan JP, Jones JC (2020). MIFlowCyt-EV: a framework for standardized reporting of extracellular vesicle flow cytometry experiments. J Extracell Vesicles.

[121] Panagopoulou MS, Wark AW, Birch DJS, Gregory CD (2020). Phenotypic analysis of extracellular vesicles: a review on the applications of fluorescence. J Extracell Vesicles.

[122] Botha J, Pugsley HR, Handberg A (2021). Conventional, high-resolution and imaging flow cytometry: benchmarking performance in characterisation of extracellular vesicles. Biomedicines.

[123] Koliha N, Wiencek Y, Heider U, Jüngst C, Kladt N, Krauthäuser S, Johnston ICD, Bosio A, Schauss A, Wild S (2016). A novel multiplex bead-based platform highlights the diversity of extracellular vesicles. J Extracell Vesicles.

[124] Kornek M, Lynch M, Mehta SH, Lai M, Exley M, Afdhal NH, Schuppan D (2012). Circulating microparticles as disease-specific biomarkers of severity of inflammation in patients with hepatitis C or nonalcoholic steatohepatitis. Gastroenterology.

[125] Julich-Haertel H, Urban SK, Krawczyk M, Willms A, Jankowski K, Patkowski W, Kruk B, Krasnodębski M, Ligocka J, Schwab R, Richardsen I, Schaaf S, Klein A, Gehlert S, Sänger H, Casper M, Banales JM, Schuppan D, Milkiewicz P, Lammert F, Krawczyk M, Lukacs-Kornek V, Kornek M (2017). Cancer-associated circulating large extracellular vesicles in cholangiocarcinoma and hepatocellular carcinoma. J Hepatol.

[126] Abbate V, Marcantoni M, Giuliante F, Vecchio FM, Gatto I, Mele C, Saviano A, Arciuolo D, Gaetani E, Ferrari MC, Giarretta I, Ardito F, Riccardi L, Nicoletti A, Ponziani FR, Gasbarrini A, Pompili M, Pola R (2017). HepPar1-positive circulating microparticles are increased in subjects with hepatocellular carcinoma and predict early recurrence after liver resection. Int J Mol Sci.

[127] Chiriacò MS, Bianco M, Nigro A, Primiceri E, Ferrara F, Romano A, Quattrini A, Furlan R, Arima V, Maruccio G (2018). Lab-on-chip for exosomes and microvesicles detection and characterization. Sensors.

[128] Kanwar SS, Dunlay CJ, Simeone DM, Nagrath S (2014). Microfluidic device (ExoChip) for on-chip isolation, quantification and characterization of circulating exosomes. Lab Chip.

[129] Shao H, Im H, Castro CM, Breakefield X, Weissleder R, Lee H (2018). New technologies for analysis of extracellular vesicles. Chem Rev.

[130] Liang K, Liu F, Fan J, Sun D, Liu C, Lyon CJ, Bernard DW, Li Y, Yokoi K, Katz MH, Koay EJ, Zhao Z, Hu Y (2017). Nanoplasmonic quantification of tumor-derived extracellular vesicles in plasma microsamples for diagnosis and treatment monitoring. Nat Biomed Eng.

[131] Rega-Kaun G, Ritzel D, Kaun C, Ebenbauer B, Thaler B, Prager M, Demyanets S, Wojta J, Hohensinner PJ (2019). Changes of circulating extracellular vesicles from the liver after Roux-en-Y bariatric surgery. Int J Mol Sci.

